# Structural Insights and Docking Analysis of Adamantane-Linked 1,2,4-Triazole Derivatives as Potential 11β-HSD1 Inhibitors

**DOI:** 10.3390/molecules26175335

**Published:** 2021-09-02

**Authors:** Doaa A. Osman, Mario A. Macías, Lamya H. Al-Wahaibi, Nora H. Al-Shaalan, Luke S. Zondagh, Jacques Joubert, Santiago Garcia-Granda, Ali A. El-Emam

**Affiliations:** 1 Department of Physical and Analytical Chemistry, Faculty of Chemistry, Oviedo University-CINN, 33006 Oviedo, Spain; UO260613@uniovi.es (D.A.O.); sgg@uniovi.es (S.G.-G.); 2 Crystallography and Chemistry of Materials, CrisQuimMat, Department of Chemistry, Universidad de Los Andes, Carrera 1 No. 18A-10, Bogotá 111711, Colombia; ma.maciasl@uniandes.edu.co; 3 Department of Chemistry, College of Sciences, Princess Nourah bint Abdulrahman University, Riyadh 11671, Saudi Arabia; nhalshaalan@pnu.edu.sa; 4 Pharmaceutical Chemistry, School of Pharmacy, University of the Western Cape, Private Bag X17, Bellville 7535, South Africa; lzondagh@uwc.ac.za (L.S.Z.); jjoubert@uwc.ac.za (J.J.); 5 Department of Medicinal Chemistry, Faculty of Pharmacy, Mansoura University, Mansoura 35516, Egypt

**Keywords:** adamantane, 1,2,4-triazole, single crystal X-ray, Hirshfeld surface analysis, molecular docking, ADME, 11β-HSD1 inhibitors

## Abstract

The solid-state structural analysis and docking studies of three adamantane-linked 1,2,4-triazole derivatives are presented. Crystal structure analyses revealed that compound **2** crystallizes in the triclinic *P*-1 space group, while compounds **1** and **3** crystallize in the same monoclinic *P*2_1_/*c* space group. Since the only difference between them is the *para* substitution on the aryl group, the electronic nature of these NO_2_ and halogen groups seems to have no influence over the formation of the solid. However, a probable correlation with the size of the groups is not discarded due to the similar intermolecular disposition between the NO_2_/Cl substituted molecules. Despite the similarities, CE-B3LYP energy model calculations show that pairwise interaction energies vary between them, and therefore the total packing energy is affected. HOMO-LUMO calculated energies show that the NO_2_ group influences the reactivity properties characterizing the molecule as soft and with the best disposition to accept electrons. Further, in silico studies predicted that the compounds might be able to inhibit the 11β-HSD1 enzyme, which is implicated in obesity and diabetes. Self- and cross-docking experiments revealed that a number of non-native 11β-HSD1 inhibitors were able to accurately dock within the 11β-HSD1 X-ray structure *4C7J*. The molecular docking of the adamantane-linked 1,2,4-triazoles have similar predicted binding affinity scores compared to the *4C7J* native ligand 4YQ. However, they were unable to form interactions with key active site residues. Based on these docking results, a series of potentially improved compounds were designed using computer aided drug design tools. The docking results of the new compounds showed similar predicted 11β-HSD1 binding affinity scores as well as interactions to a known potent 11β-HSD1 inhibitor.

## 1. Introduction

Significant attention has been devoted to adamantane-based derivatives which have long been identified for their assorted biological properties [1,2,3]. Adamantane-based drugs are presently used as efficient chemotherapies as antiviral [4,5,6], anti-TB [7,8] and anticancer [9,10,11] agents.

**Figure 1:**
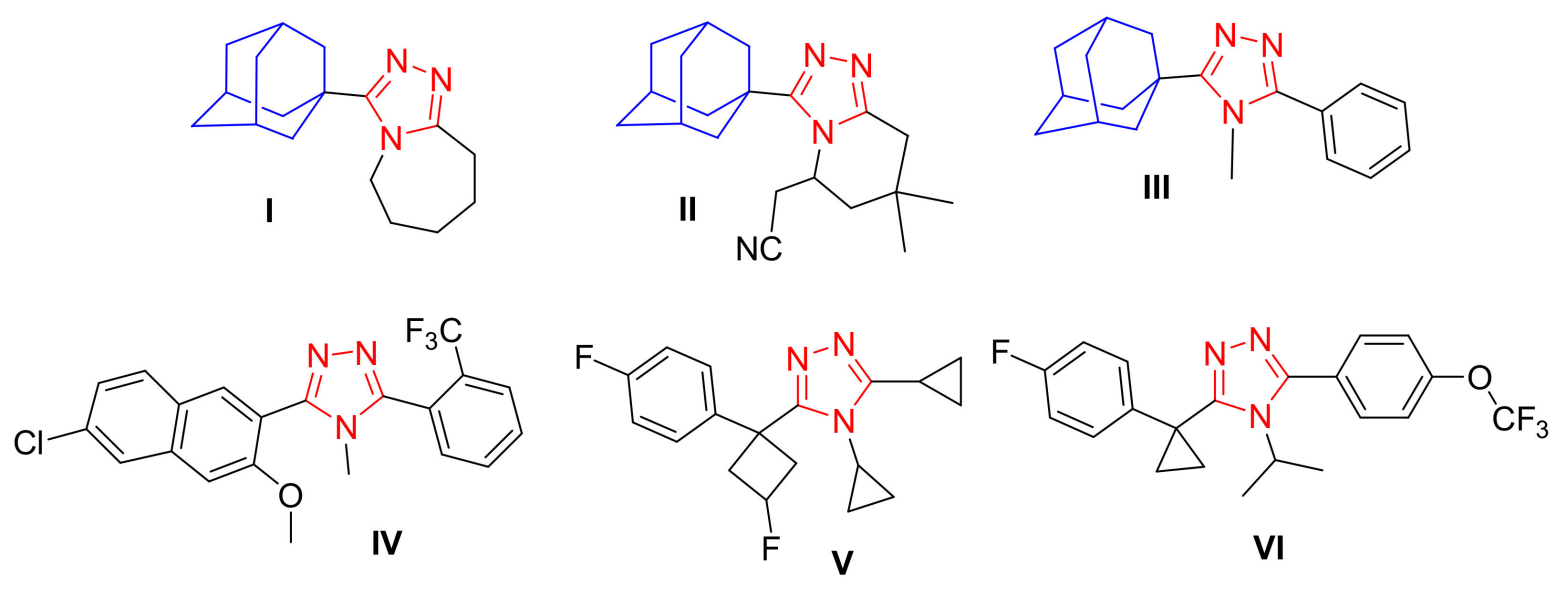
The structures the adamantyl-1,2,4-triazoles (**I**–**III**) and non-adamantyl-1,2,4-triazoles (**IV**–**VI**) 11β-HSD1 inhibitors.

The dipeptidyl peptidase IV (DPP-IV) adamantane-based drugs vildagliptin [12] and saxagliptin [13] are currently used as oral hypoglycemic agents for the treatment of type 2 diabetes. The adamantane-linked 1,2,4-triazole derivatives **I**, **II** and **III** were discovered as potent inhibitors of 11β-hydroxysteroid dehydrogenase type 1 (11β-HSD1) [14,15]. In addition, the non-adamantyl 1,2,4-triazoles **IV** [16], **V** [17] and **VI** [18] are presently under clinical investigations as 11β-HSD1 inhibitors for the treatment of type 2 diabetes and obesity (Figure 1).

**Figure 2:**
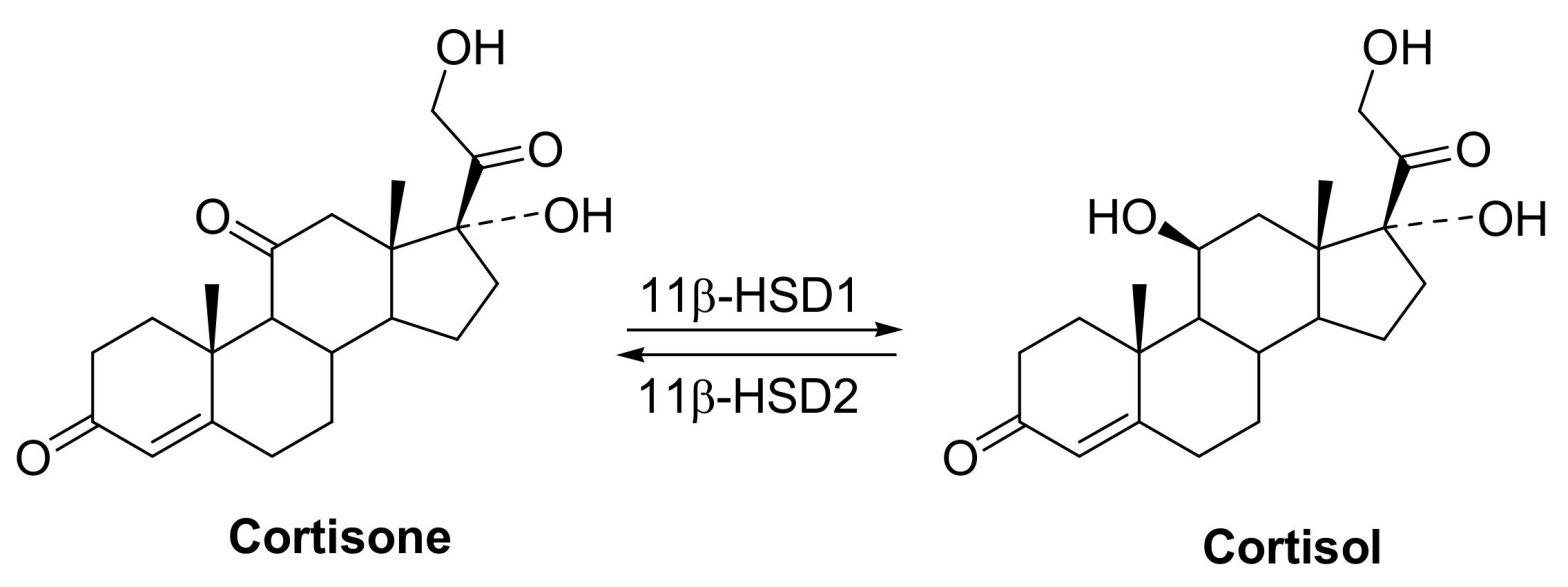
The reactions catalyzed by 11β-hydroxysteroid dehydrogenase types 1 and 2.

11β-HSD1 is an endoplasmic reticulum-associated enzyme that acts as NADPH-dependent reductase, it converts inactive cortisone to the active glucocorticoid cortisol. Cortisol stimulates gluconeogenesis through upregulation of enzymes such as phosphoenolpyruvate carboxykinase and glucose-6-phosphatase, and in adipose tissues, cortisol promotes adipogenesis and lipolysis. Thus, 11β-HSD1 regulates intracellular cortisol level and has been implicated in a number of metabolic sequelae of increased glucocorticoid tone such as visceral adiposity, elevated blood pressure, elevated fasting glucose, and dyslipidemia [19]. In contrast, the structurally related 11β-HSD2 is a NAD-dependent dehydrogenase that catalyzes inactivation of cortisol by conversion to cortisone. 11β-HSD2 is expressed in cells that contain the mineralocorticoid receptors and protects the mineralocorticoids from illicit occupation by cortisol. Inhibition of 11β-HSD2 is known to result in hypokalemia, sodium retention, and hypertension (Figure 2). Thus, the development of selective 11β-HSD1 inhibitors would be an important therapy for non-insulin-dependent diabetes, hyperglycemia, obesity, insulin resistance, hyperlipidemia, hypertension and other symptoms associated with excessive body cortisol [20,21,22].

In continuation of ongoing interest in the structural studies and potential biological applications of adamantane-based derivatives [23,24,25,26,27,28], we report herein the crystal structure, Hirshfeld surface analysis, pairwise interaction energies and electronic properties of three adamantane-linked triazole derivatives **1**–**3**. Molecular docking experiments at the 11β-HSD1 active site were also performed in order to predict the potential 11β-HSD1 binding affinity and binding interactions of the compounds.

## 2. Results and Discussion

### 2.1. Synthesis and Crystallization

**Scheme 1:**
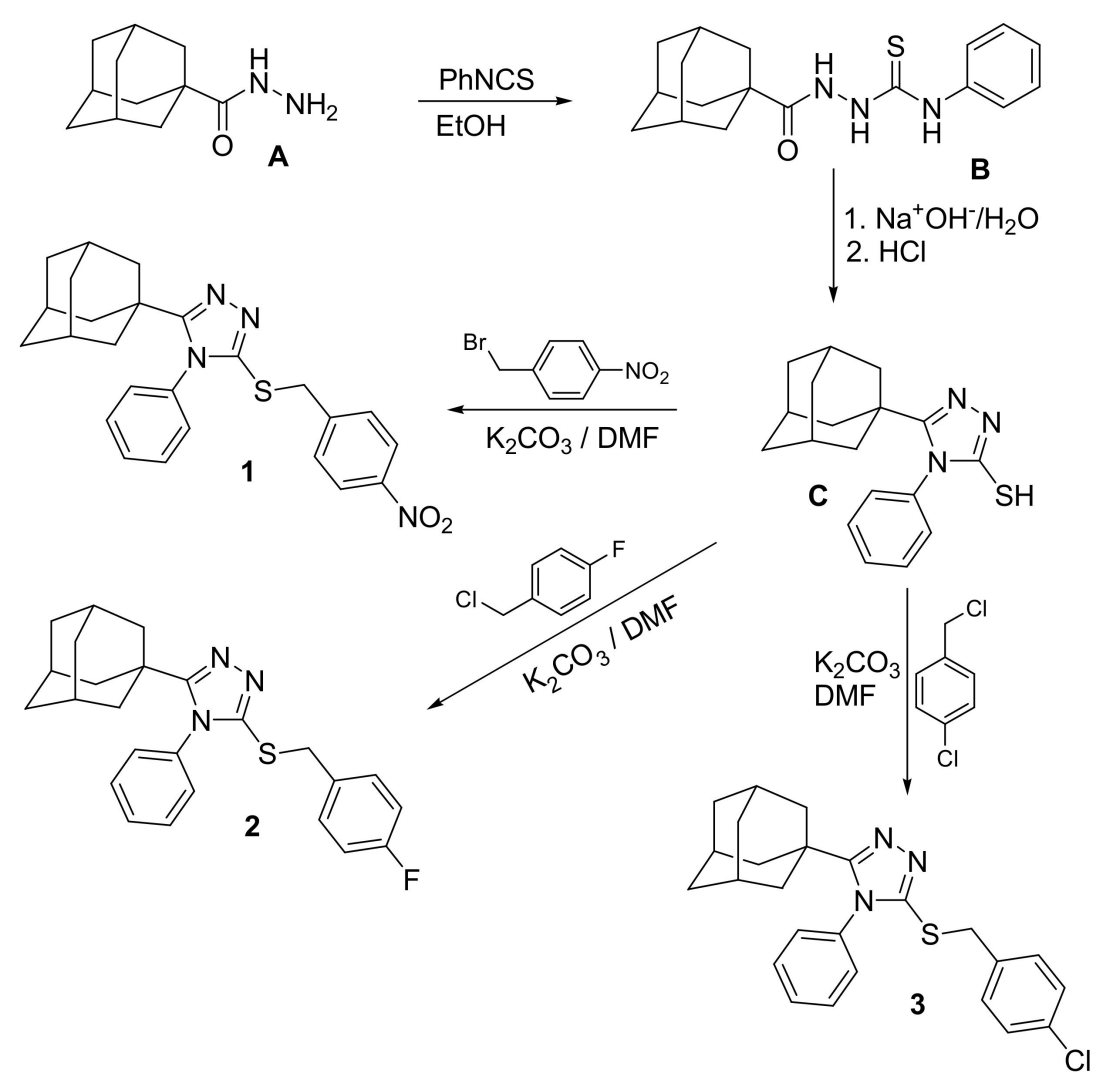
Synthesis of compounds **1**, **2** and **3**.

Compounds **1**, **2** and **3** were prepared as outlined in Scheme 1 [28], starting with adamantane-1-carbohydrazide **A** via reaction with phenyl isothiocyanate to yield the thiosemicarbazide analogue **B**, which was cyclized to the triazole analogue **C**. Compound **C** was subsequently reacted with 4-nitrobenzyl bromide, 4-fluorobenzyl chloride or 4-chlorobenzyl chloride in *N*,*N*-dimethylformamide (DMF) in the presence of potassium carbonate to yield the target compounds **1**–**3**. Single crystals suitable for X-ray diffraction were obtained by slow evaporation of a solution of the compounds in EtOH/CHCl_3_ (1:2, *v*/*v*) at room temperature.

### 2.2. Crystal Structures

**Figure 3:**
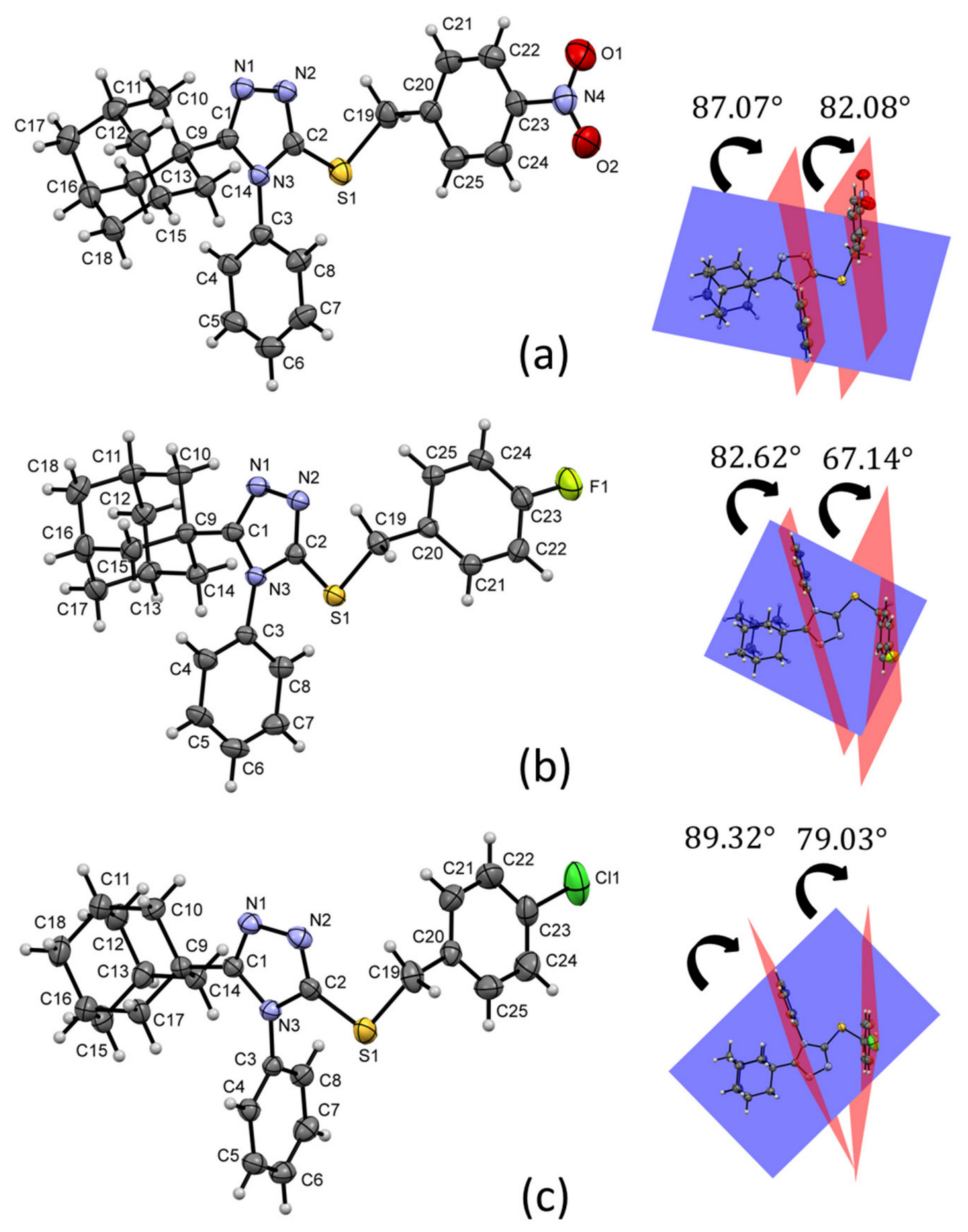
Molecular structures and dihedral angles of compounds **1** (**a**), **2** (**b**) and **3** (**c**) with anisotropic thermal vibration ellipsoids drawn at the 50% probability level.

Crystal data, data collection, and structure refinement details of compounds **1**–**3** are summarized in Table 1. The structures of the three compounds consist of three basic fragments; the adamantane cage, the triazole ring and the arylmethylsulfanyl moiety (Figure 3). A search in the CSD database version 5.41 (1 November 2019 with three updates; accessed on 1 November 2020), through the ConQuest software version 2020.2.0, for molecules with a similar core gave four related structures [29,30,31,32], but the crystal structures of compounds **1**, **2** and **3** have not been reported.

Figure 3 shows the weighted least-squares mean planes that contain the planar fragments of the molecules. The dihedral angles show a change in the molecular conformations of the aryl rings with respect to the triazole-sulfanyl fragment. In the nitrobenzyl analogue **1**, there is a tendency to the orthogonality between these groups. Meanwhile, in the halobenzyl derivatives **2** and **3**, a clear deviation was observed. The orthogonality between aryl ring is influenced by steric effects between C3/C8 and C20/C25 aryl rings and the adamantane ring.

In the two related molecules 5-(adamantan-1-yl)-3-(benzylsulfanyl)- 4-methyl-4*H*-1,2,4-triazole [29], and 5-(adamantan-1-yl)-3-[(4-chlorobenzyl)- sulfanyl]-4-methyl-4*H*-1,2,4-triazole [30], the equivalent to the C20/C25 ring is parallel to the triazole-sulfanyl fragment due to the absence of an aromatic ring in the N3 position of the triazole group. However, observing the conformational behavior in 3-(adamantan-1-yl)-5-[(2-methoxyethyl)sulfanyl]-4-phenyl-4*H*-1,2,4-triazole [31], a phenyl ring in N3 would constantly be orthogonal due to its steric effect with the adamantane cage.

**Figure 4:**
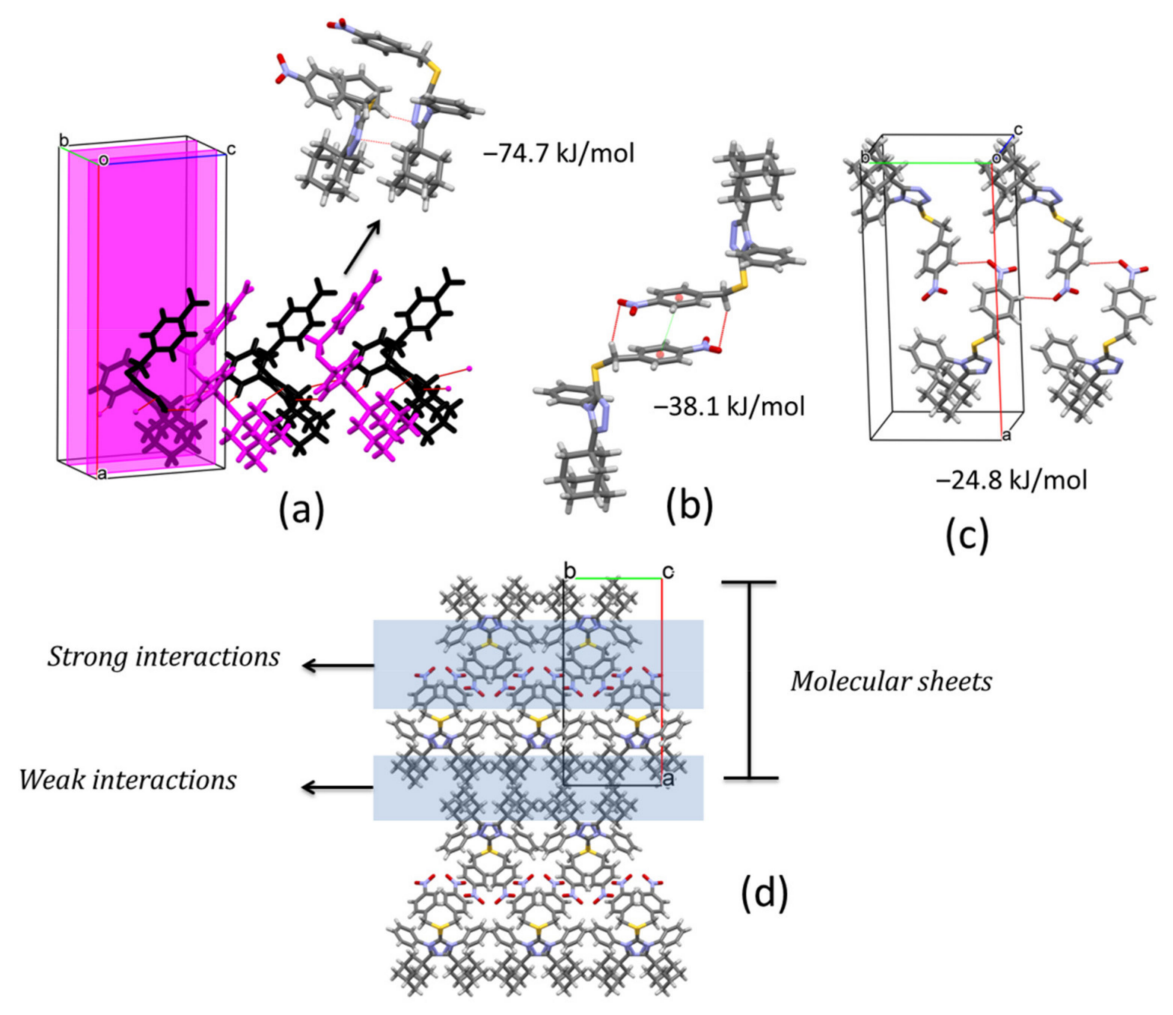
(**a**) Chain of molecules along [001] direction showing C‒H···N hydrogen-bonds. (**b**) Inversion related molecules of **1** showing C‒H···O and π···π interactions. (**c**) Molecules of **2** along [010] direction joined by C‒H···O hydrogen-bonds. (**d**) Molecular packing of **3**. Total pairwise interactions energies are shown.

In the crystal structure of compound **1**, pairs of C4‒H4···N1^i^ and C14‒H14B···N2^ii^ (symmetry codes: (i) x, 1/2 − y, −1/2 + z, (ii) x, 1/2 − y, 1/2 + z) hydrogen bonds connect molecules along [001] direction, related by a glide plane perpendicular to [010], and with distances between their molecular centroids (mean atomic position) of 5.09 Å (Figure 4a, Table 2). This interaction is the strongest contact in the crystal with a total pairwise interaction energy of −74.7 kJ/mol, being the dispersion force the principal contributor (E_elec_ = −25.9 kJ/mol; E_pol_ = −11.6 kJ/mol; E_dis_ = −93.9 kJ/mol; E_rep_ = 69.7 kJ/mol) (Table 3). The neighboring chains are connected along *a* axis through a combination of C19‒H19A···O1^iii^ and π···π^iii^ (symmetry code: (iii) 1 − x, −y, 1 − z) interactions linking inversion related molecules (Figure 4b, Table 2) with total pairwise interaction energy of −38.1 kJ/mol (E_elec_ = −13.9 kJ/mol; E_pol_ = −2.3 kJ/mol; E_dis_ = −40.8 kJ/mol; E_rep_ = 22.6 kJ/mol) (Table 3) and distances between centroids of 12.53 Å. Along [010] direction, C22‒H22···O2^iv^ (symmetry code: (iv) 1 − x, −1/2 + y, 3/2 − z) hydrogen bonds complement the formation of (100) sheets stacked along *a* axis (Figure 4c,d). The total pairwise interaction energy in this contact involving the nitro group is −24.8 kJ/mol (E_elec_ = −14.3 kJ/mol; E_pol_ = −3.7 kJ/mol; E_dis_ = −14.2 kJ/mol; E_rep_ = 8.8 kJ/mol) (Table 3). The packing showed that between molecular sheets, van de Waals forces act to maintain the three-dimensional architecture through dispersion interactions between layers of adamantane rings (Figure 4d). These contacts are rather weak with total pairwise interaction energies of −9.0 kJ/mol and −14.1 kJ/mol, maintaining larger distances (13–15 Å).

**Figure 5:**
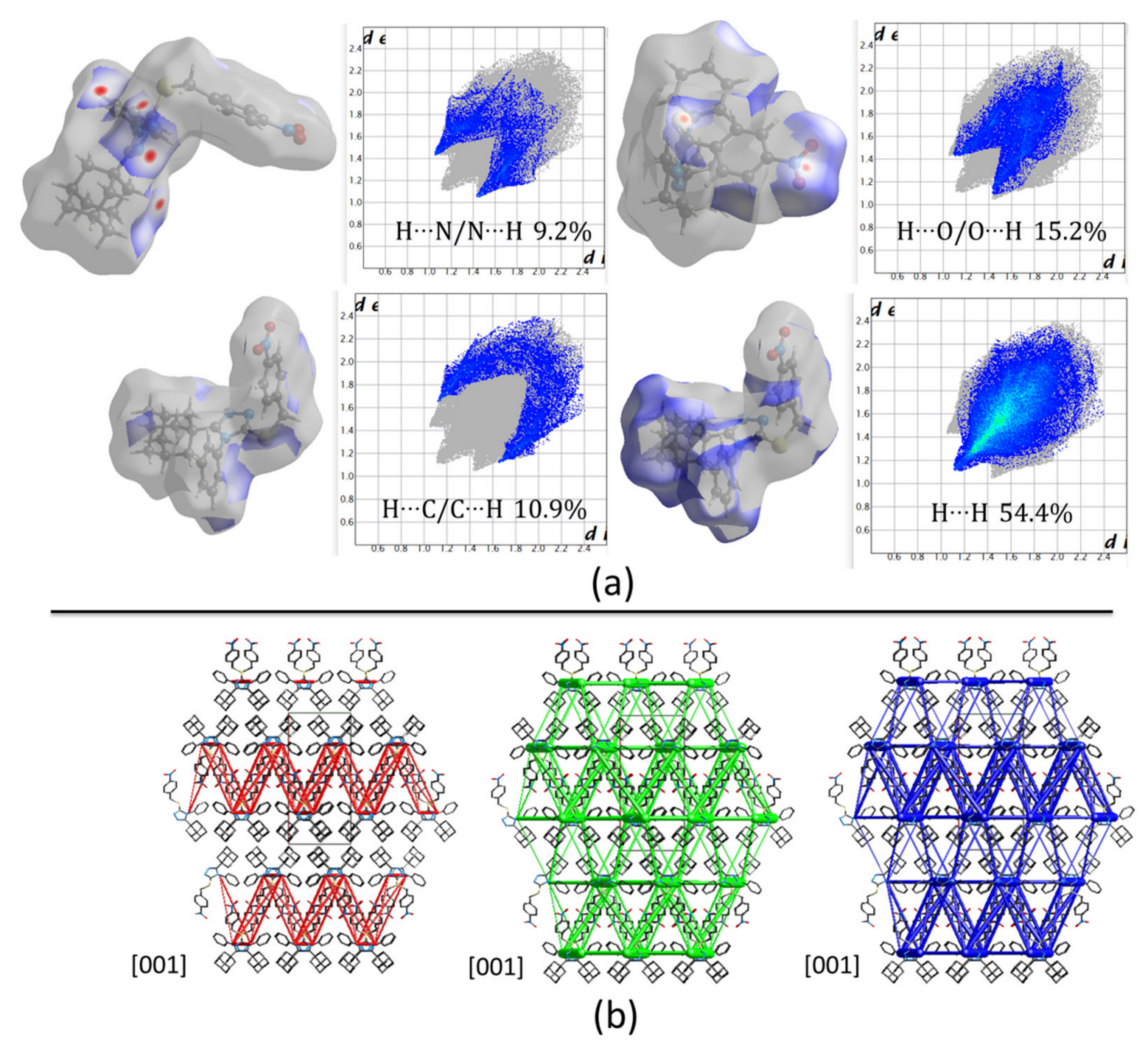
(**a**) Hirshfeld surfaces mapped over d_norm_ and the two-dimensional fingerprint plots, (**b**) Energy framework diagrams for electrostatic (red) and dispersion (green) contributions to the total interaction energies (blue) in compound **1**.

Hirshfeld surface (HFs) maps calculated at the B3LYP/6-31G(d,p) level of theory (Figure 5a) show that the shortest contacts correspond to the C‒H···(N, O) hydrogen interactions with H···N/N···H and H···O/O···H comprising 9.2% and 15.2% of the total HFs maps. The non-covalent H···H interactions occupy 54.4% of the total HFs maps showing high participation of adamantane rings in the crystal structure (Figure 4d and Figure 5a).

Computed energies between molecular pairs are represented using cylinders joining the centroids (molecular center of mass) of the molecules, with a radius proportional to the magnitude of the interaction managing a minimal cut-off of 5 kJ/mol. Figure 5b shows the energy framework diagrams for pairs of molecules for separate electrostatic (red) and dispersion (green) contributions to the total nearest-neighbor pairwise interaction energies (blue). As it is observed, the electrostatic forces act mainly along the center of the defined unit cell and define molecular sheets (Figure 4d and Figure 5b). However, dispersion forces act, not only between adamantane rings, but also in the complete structure. Despite the three-dimensional tendency of the dispersion forces, the total energy framework (blue in Figure 5b) shows a laminar energetic topology due to the strong contribution of electrostatic forces.

**Figure 6:**
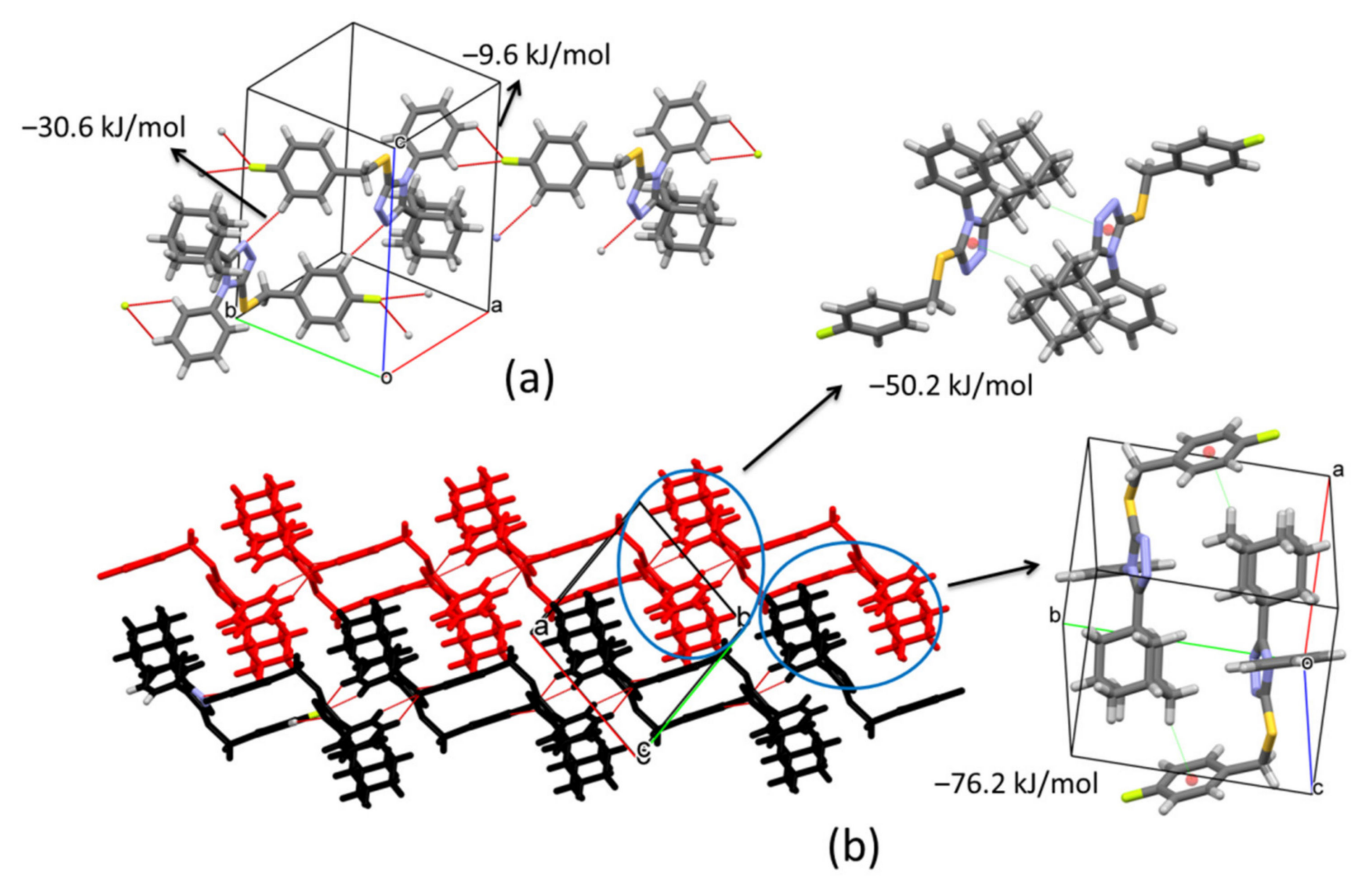
(**a**) C‒H···N and C‒H···F hydrogen-bonds joining molecules along [1–10] direction. (**b**) C‒H···π interactions involved in the formation of (001) sheets.

The supramolecular structure in compound **2** changes compared to compound **1**. In this case, the presence of the fluorine atom (F) on the aryl ring varies the connection between molecules. Inversion related molecules are linked by pairs of C24‒H24···N1^v^ (symmetry code: (v) −x, 1 − y, 1 − z) hydrogen bonds forming dimers with distances between their molecular centroids (mean atomic position) of 8.99 Å (Figure 6a, Table 2). The total pairwise interaction energy that keeps the dimers connected has a value of −30.6 kJ/mol (E_elec_ = −15.9 kJ/mol; E_pol_ = −7.5 kJ/mol; E_dis_ = −27.7 kJ/mol; E_rep_ = 25.6 kJ/mol) (Table 3). These pairs of inversion related molecules are further connected by bifurcated C4‒H4···F1^vi^ and C5‒H5···F1^vi^ (symmetry code: (vi) 1 + x, −1 + y, z) hydrogen interactions to build chains along [1–10] direction (Figure 6a, Table 2). The connection between dimers through F atoms shows weaker total interaction energy, −9.6 kJ/mol, manifested in the distance between centroids (12.53 Å). Inside chains, the molecular disposition allows the formation of C15‒H15B···Cg2^vii^ (symmetry code: (vii) 1 − x, −y, 1 − z) contacts with a total interaction energy of −50.2 kJ/mol (E_elec_ = −14.2 kJ/mol; E_pol_ = −4.2 kJ/mol; E_dis_ = −59.8 kJ/mol; E_rep_ = 32.4 kJ/mol) (Figure 6b, Table 2 and Table 3). The high value of the dispersion term suggests a strong participation of the adamantane rings. Neighboring chains are connected by C12‒H12A···Cg1^viii^ (symmetry code: (viii) 1 − x, 1 − y, 1 − z) interactions along *b* axis forming (001) sheets which correspond to the strongest contacts in the crystal with a total energy of −76.2 kJ/mol (E_elec_ = −15.3 kJ/mol; E_pol_ = −5.8 kJ/mol; E_dis_ = −101.8 kJ/mol; E_rep_ = 53.3 kJ/mol) (Figure 6b, Table 2 and Table 3). These interactions involve the highest dispersion energy in the solid as a result of the short distance between molecules (5.63 Å) that allows an important closeness between adamantane rings (6.80 Å between their centroids) (Figure 6b).

**Figure 7:**
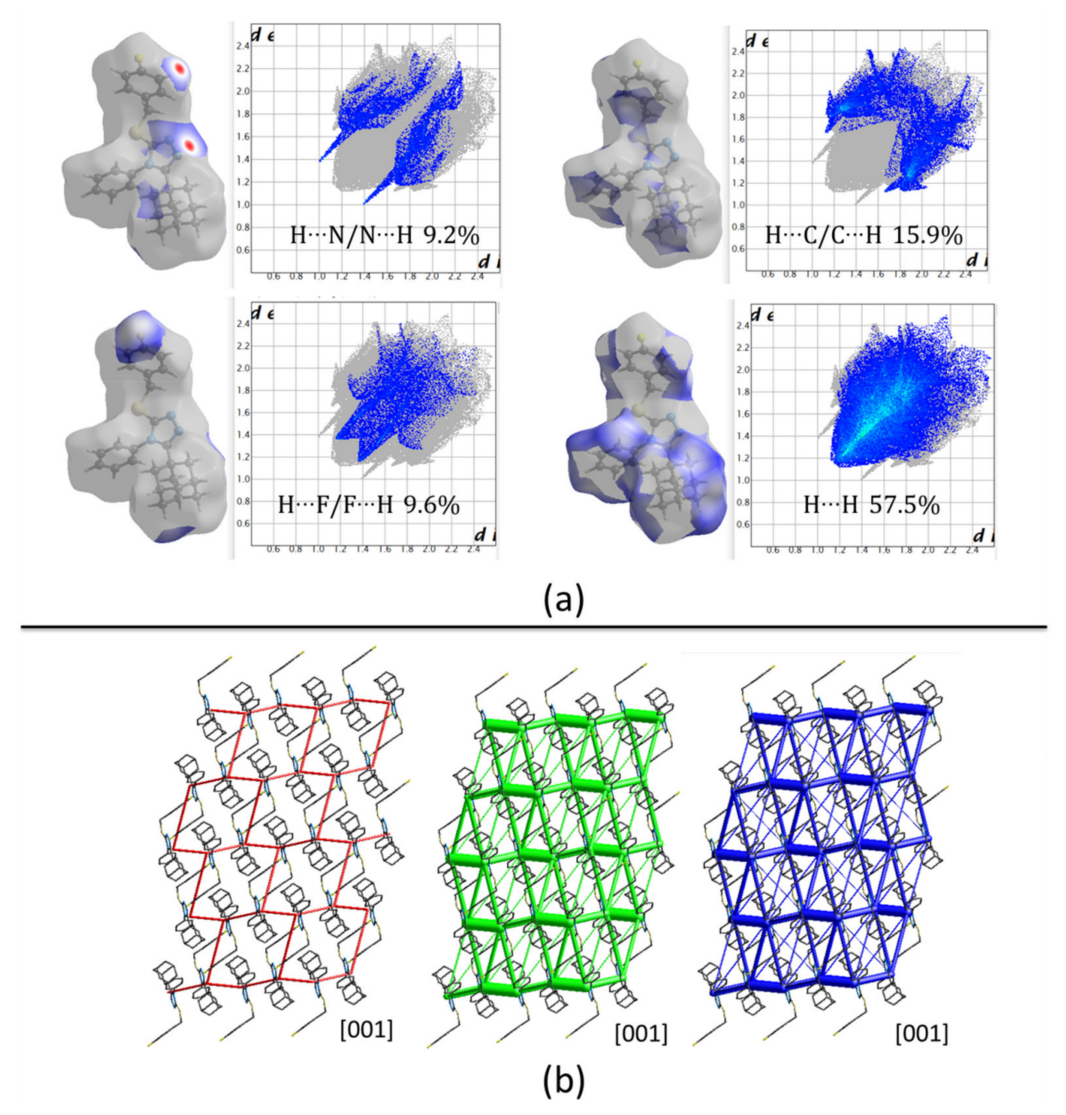
(**a**) Hirshfeld surfaces mapped over d_norm_ and the two-dimensional fingerprint plots, (**b**) Energy framework diagrams for electrostatic (red) and dispersion (green) contributions to the total interaction energies (blue) in compound **2**.

Hirshfeld surfaces (HFs) mapped over d_norm_ only present two red spots correlated to C24‒H24···N1^v^ interactions with H···N distances of 2.56 Å. The rest of the surface shows blue/white colors, representing long contacts with non-covalent H···H interactions covering 57.5% of the total surface (Figure 7a). The energy framework diagrams show that dispersion forces (green) act with a three-dimensional topology. However, the cylinders joining adamantane rings have higher radius as a consequence of the high dispersion forces acting between them (Figure 7b) showing the importance of these rings in the formation of the crystal.

**Figure 8:**
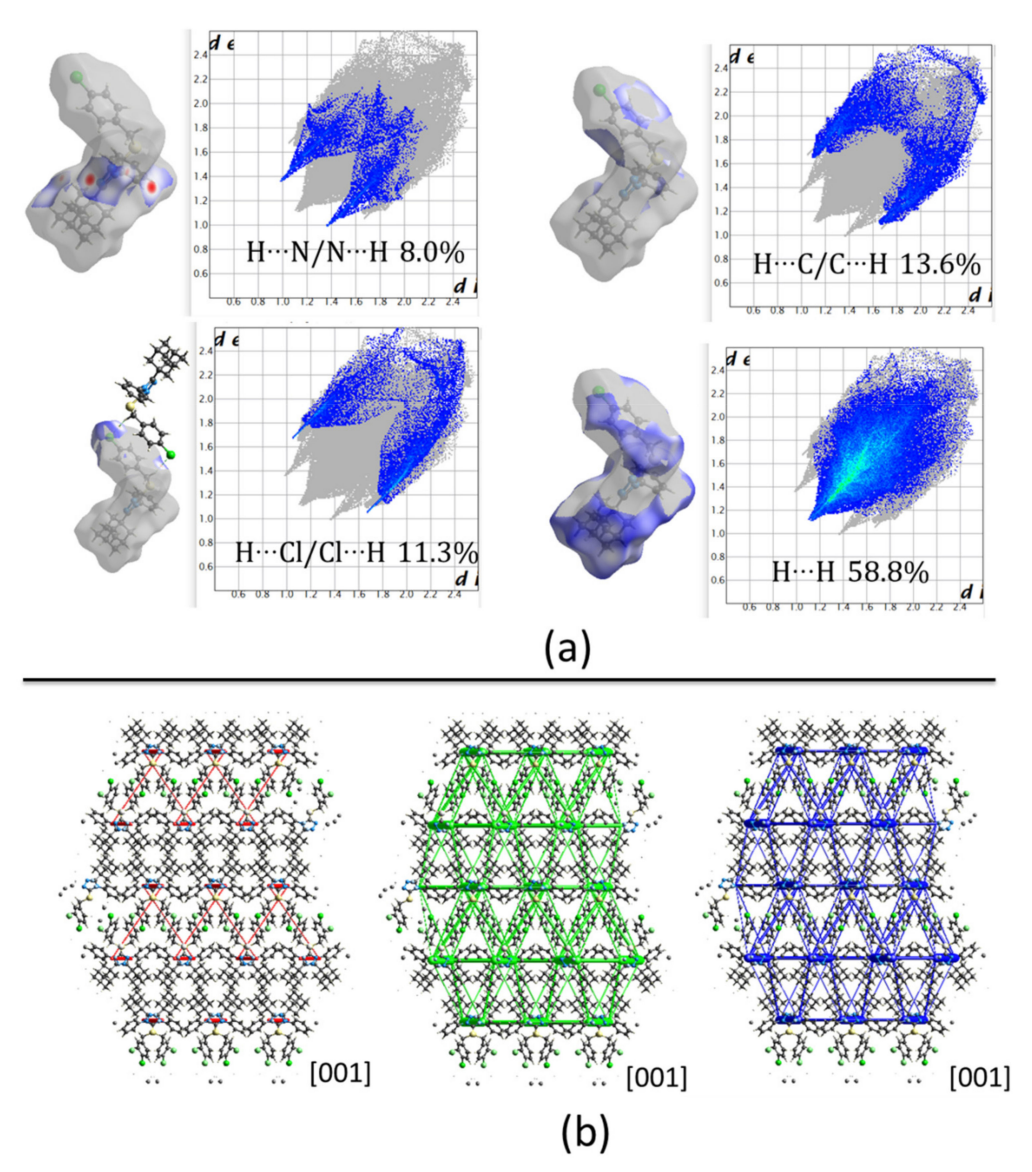
(**a**) Hirshfeld surfaces mapped over d_norm_ and the two-dimensional fingerprint plots, (**b**) Energy framework diagrams for electrostatic (red) and dispersion (green) contributions to the total interaction energies (blue) in compound **3**.

Compound **3** crystallizes in a very similar form as **1**, sharing the same monoclinic *P*2_1_/*c* space group and cell parameters with comparable dimensions. As mentioned above for compound **1**, in this case, also pairs of C4‒H4···N1^i^ and C14‒H14A···N2^ii^ (symmetry codes: (i) x, 1/2 − y, −1/2 + z, (ii) x, 1/2 − y, 1/2 + z) hydrogen bonds link molecules to form chains along [001] direction (See Figure 4 for reference). The centroids are separated by 5.24 Å and the total pairwise interaction energy is −72.4 kJ/mol (E_elec_ = −29.8 kJ/mol; E_pol_ = −11.5 kJ/mol; E_dis_ = −84.8 kJ/mol; E_rep_ = 67.1 kJ/mol) (Table 2 and Table 3). The high structural similarity is also observed in the C19‒H19B···Cl1^iii^ and π···π^iii^ (symmetry code: (iii) 1 − x, −y, 1 − z) stacking interactions joining molecules separated by 12.67 Å and with total pairwise interaction energy −25.2 kJ/mol (E_elec_ = −11.0 kJ/mol; E_pol_ = −1.5 kJ/mol; E_dis_ = −32.3 kJ/mol; E_rep_ = 25.5 kJ/mol) (Table 2 and Table 3). This value is lower compared with the same pair of molecules in compound **1** which is an indication of weaker attractions due to the change of a nitro group by a halogen. In general, the packing in compound **3** is similar to **1**. Nevertheless, C‒H···O hydrogen bonds involving the nitro group in compound **1** induce a different energy framework compared to compound **3** (Figure 8b), being the contribution of electrostatic forces lower in the last case. Also, the 2D fingerprints show differences between both structures. In the present case, the spikes from H···N interactions are most prominent and sharped in compound **3** which is consequent with shortest distances in C4‒H4···N1^i^ (Figure 8a).

**Figure 9:**
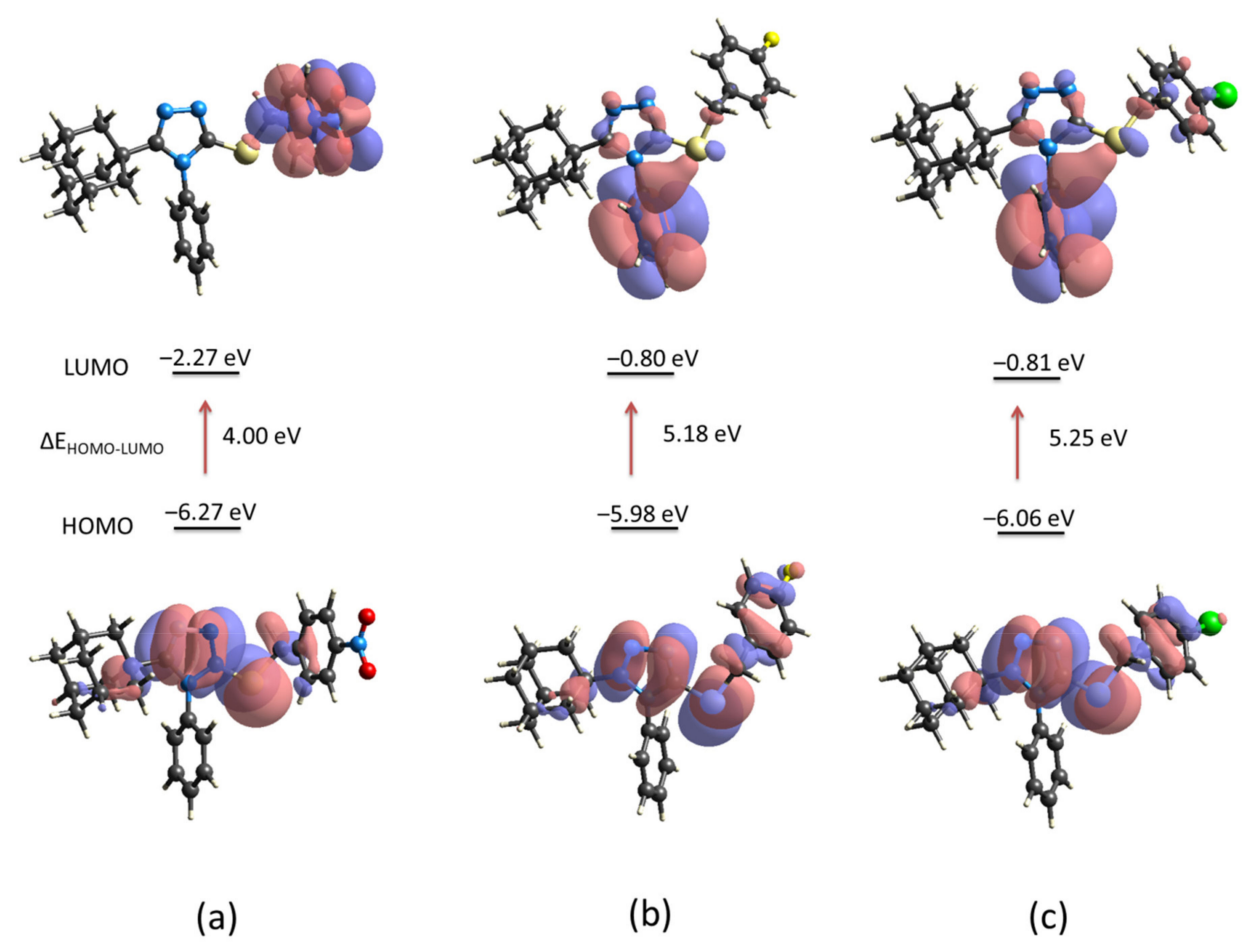
Computed energy levels HOMO–LUMO for compounds (**a**) **1**, (**b**) **2** and (**c**) **3**, using the crystallographic information files (cif) by B3LYP method using the 6-31G(d,p) basis set.

The energy levels of the electron-donor HOMO (highest occupied molecular orbital) and electron-acceptor LUMO (lowest unoccupied molecular orbital) for compounds **1**, **2** and **3** were computed using the crystallographic information (Figure 9). The energy gap between molecules is similar for compounds **2** and **3** but different from compound **1**. The presence of the nitro group (NO_2_) in the *para* position of the aryl group in compound **1** induces the charge-transfer interaction involves mainly the triazole-sulfanyl and aryl (Ar-NO_2_) moieties (Figure 9a). As it is well known, the NO_2_ group is an electron withdrawing group which under the description of their resonant structures, induces a concentration of positive charge at the respective *ortho*-, *para*- positions, being *meta*-directors to electrophilic substitutions. On the other hand, halides are *ortho*-, *para*- directing groups, however, with a mildly deactivation behavior due to their dual properties as inductive withdrawal and resonance donation. This last description is the case of compounds **2** and **3**. In these molecules, the charge-transfer interaction involves the triazole-sulfanyl and Ar-halogen moieties in the HOMO level, and the triazole-sulfanyl and aryl moieties in the LUMO level (Figure 9b,c). The strong electron withdrawing property of NO_2_ group is observed in the decrease of the LUMO energy (−2.27 eV). Even, the lower energy gap in compound **1** characterizes this molecule as soft, a property associated with low kinetic stability, high chemical reactivity, and greater polarizability due to the lower energy needed for excitation [33]. Considering that HOMO and LUMO energies represent the ability to donate and gain an electron, respectively, it is clear that the presence of the NO_2_, and F/Cl groups, modify the reactivity of these molecules. In all cases, the triazole-sulfanyl fragment has an active disposition to donate electrons but the molecular disposition towards potential nucleophilic addition reactions is different in compound **1** compared with compounds **2** and **3**. Based on the calculated molecular orbitals, some derived parameters were highlighted such as chemical hardness, chemical potential and electrophilic index among others (Table 4).

The chemical potential and chemical hardness were calculated using Koopmans’ theorem as: *μ* = (E_LUMO_ + E_HOMO_)/2, and *η =* (E_LUMO_ − E_HOMO_)/2 [34,35]. The electrophilicity index was calculated according to Parr et al. as *ω = μ*^2^*/*2*η* [36]. The propensity to donate charge or electrodonating power, *ω*^−^ = [(3IP + EA)^2^ / 16(IP − EA)], the propensity to accept charge or electroaccepting power, *ω*^+^ = [(IP + 3EA)^2^ / 16(IP − EA)], and net electrophilicity Δ*ω*^±^ = *ω*^+^ + *ω*^−^, were calculated according to Gázquez et al. [37]. The magnitude of hardness *η* parameter allows concluding that, effectively, molecules of **1** could be more reactive than **2** and **3**. This softness is also observed in the lower value of the LUMO energy (−2.27 eV) which signifies that it is the best electron acceptor. This property suggests that **1** is the strongest electrophile, which is in good agreement with the higher net electrophilicity index (Δ*ω*^±^ = 9.61 eV).

### 2.3. Prediction of Activity Spectra and Molecular Docking Studies

Prediction of Activity Spectra (PASS) is an online structure-activity relationship tool that predicts pharmacological properties of over 4000 types of biological activities and targets based on the structure of the studied compound [38]. PASS analysis was therefore used to predict the pharmacological properties of compounds **1**, **2** and **3**. The results indicated that the predicted highest probability of biological activity (Pa) was for anti-obesity and anti-diabetic (type 2) activities (Table 5). The ability of compounds **1**, **2** and **3** to inhibit the 11β-HSD1 enzyme, which is a target that has been identified to potentially treat obesity and type-2 diabetes [19,20,21,22], was predicted at Pa values of 0.619 to 0.678. These predicted 11β-HSD1 inhibitory abilities fell well within the top predicted activities of each compound (Table 5).

Molecular docking has become widely used in the development of novel therapeutic agents. These techniques greatly improve our knowledge of the structural activity relationships between ligands and active site residues as well as conformational changes of the active site caused by the complex formed with a ligand [39]. Therefore, the quality of the three-dimensional (3D) protein X-ray crystal structure is crucial for reproducible and accurate ligand docking. There are several methods in determining the best protein crystal structure with the most common method considering both the resolution (Å) as well as the R-factor values [40]. Conversely, it has been reported that these metrics are not absolute and that these metrics alone cannot appropriately predict the performance of molecular docking within a specified protein crystal [41,42]. Holo X-ray crystal structures with different co-crystallized ligands affect the conformation of the receptors active site residues. Therefore, the native ligand or non-native ligands with similar molecular structures of a ligand-receptor complex will more likely exhibit tighter binding affinities compared to structurally different non-native ligands [43]. Therefore, it can be proposed that if non-native ligands are able to dock into X-ray protein structure with a similar binding pose to their crystallized binding pose within their native enzyme, then it will probably be more proficient at accurately docking experimental ligands. In this study, the holo crystal structures were selected using the resolution and R-factor scores obtained from the protein data bank (PDB) (https://www.rcsb.org, accessed on 26 July 2021) [44]. Thereafter, the native ligands were re-docked into the respective enzymes using the self-docking approach. The self-docking approach assists in validating the docking protocol as well as determining the suitability of the protein structures. Finally, the cross-docking approach was employed to dock non-native 11β-HSD1 ligands into multiple 11β-HSD1 enzymes The cross-dock approach is used to establish the most suitable X-ray crystal structure to be used for experimental ligand docking [43]. Previous studies have shown that non-native ligands with similar chemical structures to the native ligand, in the cross-docking approach, exhibited similar binding poses in reference to their crystallized binding pose [45,46]. Therefore, we extracted holo 11β-HSD1 protein structures that contained co-crystallized ligands that were either similar or diverse in chemical structure.

Sixteen holo 11β-HSD1 X-ray structures and their native ligands were prepared, docked and analyzed using the self-docking protocol and docking evaluation criteria (Table 6). Ten of the sixteen holo X-ray structures successfully re-docked the native ligands met the criteria requirements. These structures were considered for cross-docking. Protein *4C7J*, was selected as the most suitable template for superposition of the other crystal proteins active sites as it exhibited the best overall criteria results.

The successfully self-docked holo protein structures were prepared and superposed onto the X-ray crystal structure of *4C7J*. The ligands were prepared and saved in a merged database. The docking protocol was altered to 100 returned poses for re-scoring to improve docking accuracy. To ensure the validity of the docking protocol, the selfdocking criteria was used on the re-docked native ligands. Three of the ten holo X-ray structures (*3HFG*, *4C7K* and *4K1L*) were unable to reproduce the native ligand’s crystallographic binding pose and therefore, were removed from further analysis. The cross-docking results were averaged over all the ligands for comparison (Table 7 and Supplementary Materials, Tables S1–S3).

**Figure 10:**
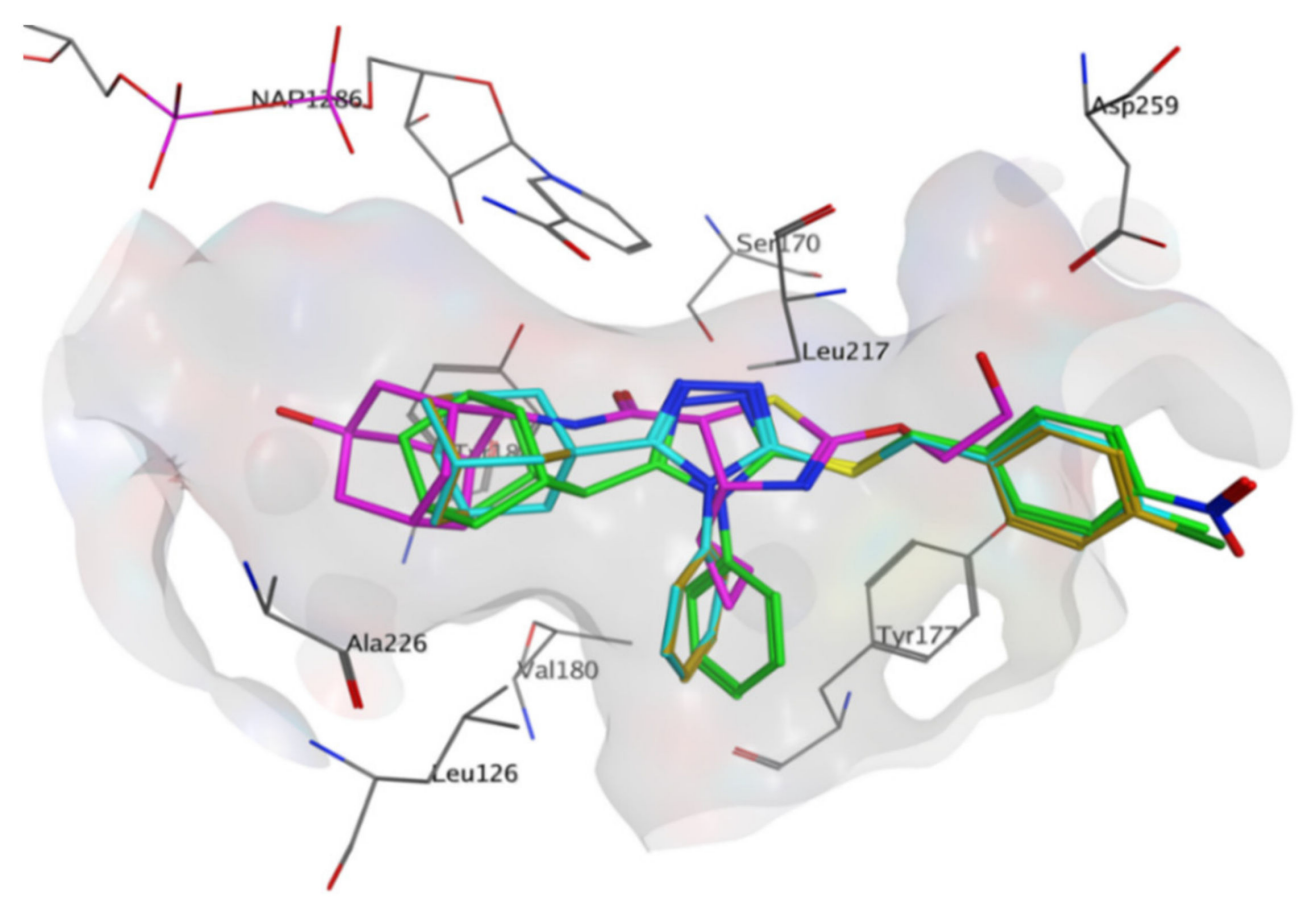
The binding orientations of **4YQ** (magenta) and compounds **1** (green), **2** (blue), **3** (green) within the 11β-HSD1 active site using MOE 2020 software suite [53]. The shift of compounds **1**–**3** towards the entrance of the active site, the exposure of the substituents of the benzylsulfanyl moiety to the external environment as well as the rotation of the triazole moiety towards the NAP co-factor and it’s out of plane nature in reference to Ser 170 and Tyr 183 was observed. Hydrogens were removed for illustration purposes.

**Figure 11:**
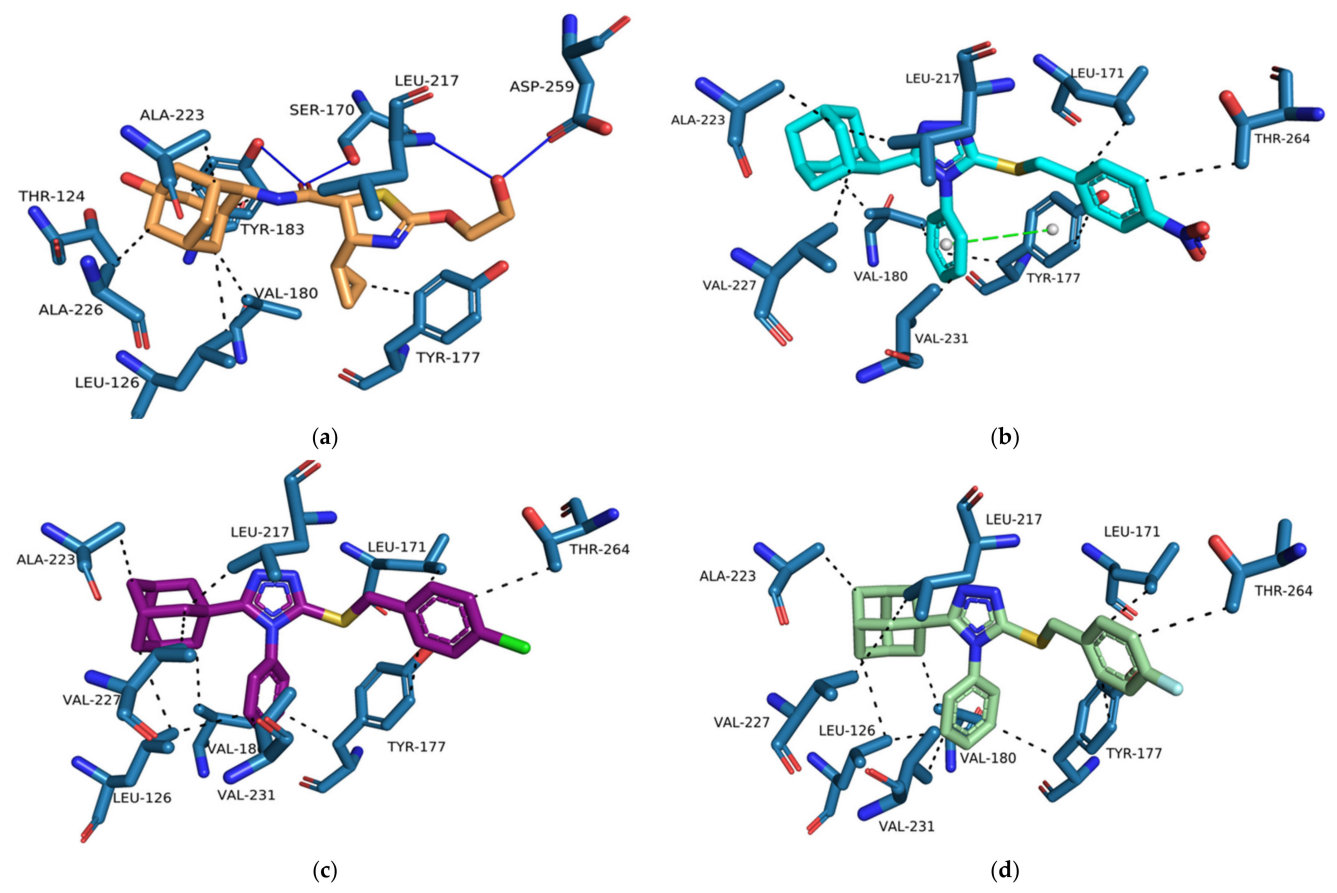
The binding orientations and interactions of **4YQ** (**a**) and compounds **1** (**b**), **2** (**c**) and **3** (**d**) within the 11β-HSD1 active site were analyzed on PLIP [48] and visualized using Pymol molecular graphics system. The hydrogen bond and hydrophobic interactions are indicated by the solid blue and black dashed lines respectively.

The importance of cross-docking was observed as multiple protein structures were unable to successfully dock non-native ligands that met the docking analysis criteria. Only *4HX5* was able to reproduce an average top pose below 2 Å, 4*C7J* and *4HX5* were able to reproduce an average lowest pose RMSD of below 2 Å, 4*C7J*, *4HX5*, *4IJV*, *4IJW* and *5QII* were able to reproduce 3 or more crystallographic poses of non-native ligands below 2 Å and no protein structures were able to reproduce a RMSD average across the top 5 poses below 3 Å. Most of the protein structures were able reproduce the binding affinity scores of the non-native ligands to within 1.0 kcal/mol when compared to the native binding affinity scores (Supplementary Materials, Table S4). The majority of the ligands exhibited non-native receptor binding affinity scores higher than the native receptor binding affinity scores. Thus, these scores confirm that ligands exhibit tighter predicted binding affinity when docked within their native receptor when compared to non-native receptors. The results also confirmed that proteins containing co-crystallized ligands with similar chemical structures exhibited RMSD results within the self-docking criteria. Protein structures 4*C7J* and *4C7K* as well as *4IJW* and *5QII* exhibited excellent RMSD cross-docking results. Even though *4C7K* was unable to reproduce self-docking RMSD values within the docking criteria it was however able to do so for 4*C7J*. Protein structures 4*C7J* and *4HX5* exhibited the best overall cross-docking results. 4*C7J* was chosen as the most appropriate protein structure for the docking of compounds **1**, **2** and **3** over *4HX5*. Compounds **1**–**3** have a number of structural features, e.g., the adamantane- and triazole moieties, that are similar to the potent 11β-HSD1 co-crystallized inhibitor 4-cyclopropyl-*N*-(trans-5-hydroxy-2-adamantyl)-2-(2-hydroxyethoxy)-thiazole-5-carboxamide (**4YQ,** 11β-HSD1 IC_50_ = 9.9 nM) [47]. *4HX5* was also unable to significantly reproduce the crystallized poses for both 4*C7J* and *4C7K* native ligands. Compounds **1**, **2** and **3** were prepared and docked into X-ray protein structure 4*C7J* as described in the methods section. The docking results were analyzed using web-based protein-ligand complex analysis server Protein-Ligand Interaction Profiler (PLIP, https://plip-tool.biotec.tu- dresden.de, accessed on 26 July 2021) [48]. Compounds **1**, **2** and **3** docked conformations were able to bind to the active site with binding affinity scores of −8.30 kcal/mol, −7.70 kcal/mol and −7.83 kcal/mol, respectively. The binding affinity scores are similar to **4YQ** (−8.20 kcal/mol). **4YQ** exhibited hydrogen bond (HB) interactions with important active site residues Ser 170 (HB), Tyr 183 (HB), Asp 259 (HB), Leu 217 (HB) (Figure 10 and Figure 11a). The adamantane moiety of **4YQ** was buried deep within the hydrophobic pocket exhibiting hydrophobic interactions (HI) with residues Ala 223, Ile 121, Val 180, Tyr 183 and Leu126. The 4-cyclopropylthiazole moiety substituent interacted with Tyr 177 with a hydrophobic interaction within the secondary entrance, positioned center bottom of the active site in Figure 10. Interactions with these residues have previously shown to be important for 11β-HSD1 inhibition [49,50,51,52].

Compounds **1**–**3** were orientated in a similar manner to **4YQ** with the adamantane moiety positioned within the hydrophobic pocket between Tyr183, Ala 226, Ala 223 and Val180, the benzylsulfanyl moiety spanning towards the primary entrance of the active site and the 4-phenyl-1,2,4-triazole moiety occupying a similar position within the secondary entrance as the 4-cyclopropylthiazole moiety of **4YQ** (Figure 10 and Figure 11). In general, compounds **1**–**3** exhibited weak hydrophobic interactions with 11β-HSD1 active site residues with only compound **1** interacting with Tyr 177 through a π-π interaction. The phenyl substitution conjugated to the triazole moiety is positioned within the secondary entrance of the active site and resulted in pulling the compound towards the primary entrance of the active site. The positioning of the phenyl substitution also caused the triazole moiety to rotate out of plane from key residues responsible for active site catalysis Ser 170 and Tyr 183 (Figure 10 and Figure 11). The lipophilic adamantane moiety is then removed from the deep pocket within the active site reducing the number of hydrophobic interactions within the hydrophobic pocket. The nitro group and halogen substitutions are exposed to the outside of the active site and therefore lacks the ability to form potential binding interactions with the active site pocket (Figure 10).

**Figure 12:**
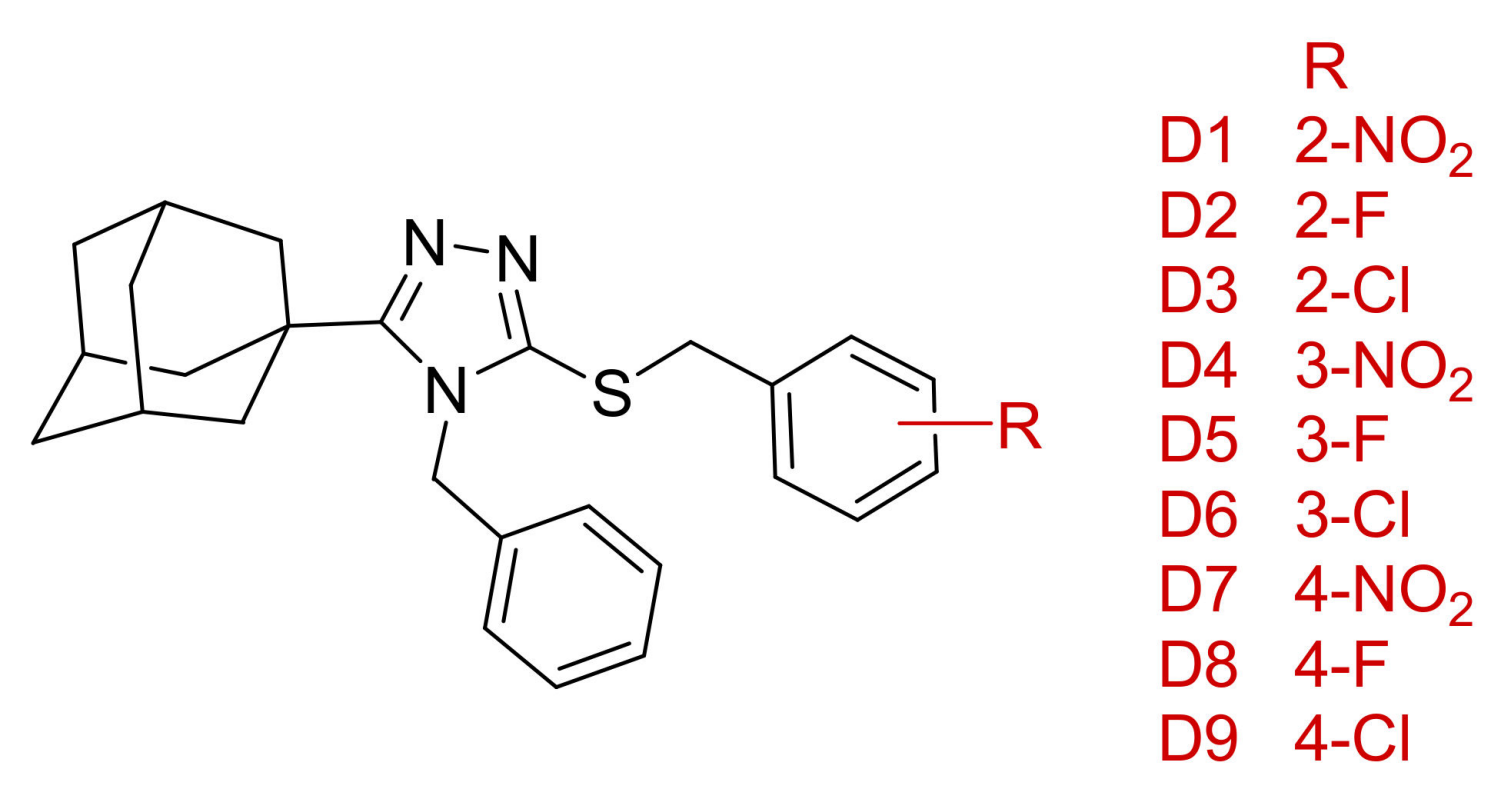
Chemical structure and R- group substitutions of the CADD designed compound series.

**Figure 13:**
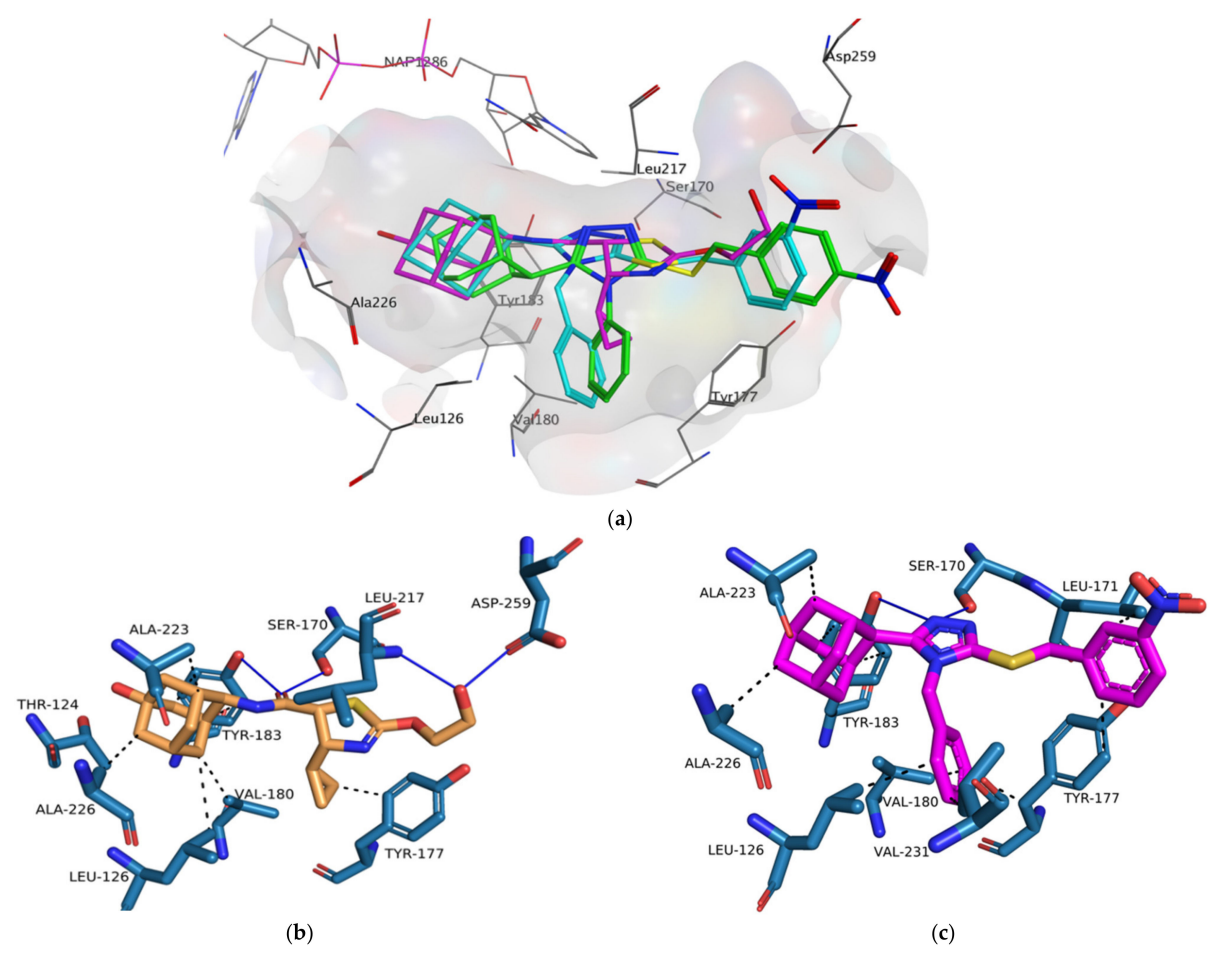

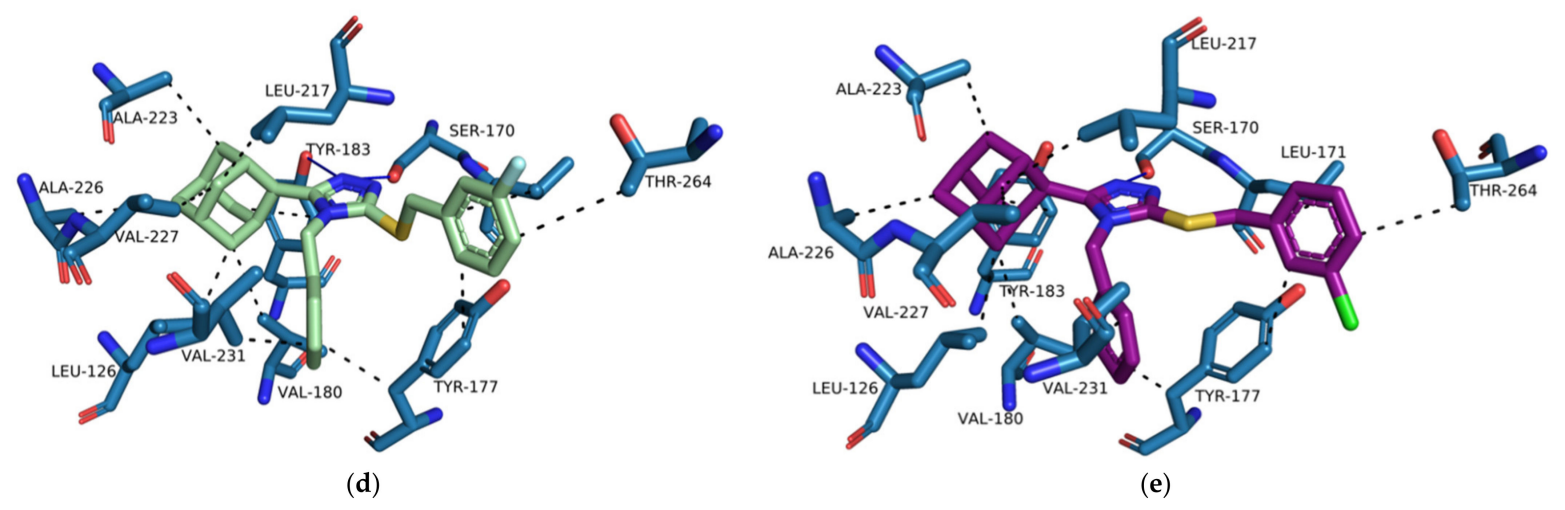
(**a**) The binding orientations of **4YQ** (magenta), compound **1** (blue) and compound **D4** (blue) within the 11β-HSD1 active site are shown using (MOE) 2020 software suite [53]. Compound **D4** has shifted deeper into the and the triazole moiety has rotated to a more planar orientation in reference to Ser 170 and Tyr 183 when compared to compound **1**. The binding orientations and interactions of **4YQ** (**b**), **D4** (**c**), **D5** (**d**) and **D6** (**e**) within the 11β-HSD1 active site are shown were analyzed on PLIP [48] and visualized using Pymol molecular graphics system [54]. The hydrogen bond and hydrophobic interactions are indicated by the solid blue and black dashed lines respectively.

Based on the docking results, we propose a series of compounds (**D1**–**D9**, Figure 12) based on the structures of compounds **1**–**3** as potential 11β-HSD1 inhibitors with improved binding interactions and binding affinity scores. The proposed compounds were designed through the employment of rational as well as computer-aided drug design (CADD) strategies. The designed compounds were structurally altered by the addition of a single carbon linker between the triazole moiety and the phenyl substitution. The additional carbon linker is designed to improve the compound flexibility and allow the triazole moiety to interact with key residues Ser 170 and Tyr 183 through hydrogen bond interactions. We also varied the positions of the nitro and halogen substitutions on the benzylsulfanyl moiety. The CADD designed compounds’ binding conformations exhibited binding affinity scores between −7.98 to −8.48 kcal/mol (Supplementary Materials, Table S5). The majority of the designed compounds interacted with both Ser 170 and Tyr 183 through hydrogen bond interactions as well as Tyr 177 and Tyr 183 through hydrophobic interactions, similar to **4YQ** (Figure 13b–e and Supplementary Materials, Tables S6–S18).

The increased flexibility of the phenyl substitution by the addition of the single carbon linker resulted in the rotation of the triazole moiety into a planar position in reference to the Ser 170 and Tyr 183 residues and pushed the lipophilic adamantane moiety deeper into the hydrophobic pocket (Figure 13a). The *para*-substituted compounds, **D7**–**D9**, were still exposed to the external environment due to the length of the structures of the compounds. The *ortho-* and *meta*-substituted compounds, **D1**–**D6**, reduced the exposure of the substitutions to the external environment. However, the halogen substitutions did not interact with the Asp 259. When halogens are bound to aromatic carbons the electron density of the halogen is redistributed resulting in the formation of an electrophilic region at the distal end of the halogen atom. The electrophilic region known as the sigma-hole contributes to the halogen bond donating capabilities of halogens [55]. However, since linearity of halogen bond donor angles is crucial for halogen bonding, analysis tool like PLIP have strict linear halogen bond donor angle cut-offs of ≥ 165° [48]. The angles between the halogen of the halogen substituted series **D** compounds and Asp 259 was consistently below 100° and therefore no halogen donor bond interactions were formed. No binding interactions were formed between the nitro substitution and Asp 259, even though they are observed to be in proximity to one another (Figure 13a). Both the nitro substitution and Asp 259 are anionized within the 11β-HSD1 active site and consequently are unable to form any type of binding interaction. Therefore, proton donors containing substitutions on the benzylsulfanyl moiety should be considered for future studies.

### 2.4. In Silico Toxicity and ADME Prediction Studies

The predicted toxicity of compounds **1**–**3** and **D1**–**D9** were calculated using the online toxicity prediction tool ProTox-II (https://tox-new.charite.de/protox II, accessed on 26 July 2021) [56] and the online absorption, distribution, metabolism and excretion (ADME) prediction tool SwissADME (http://www.swissadme.ch, accessed on 26 July 2021) [57]. ProTox-II calculate a compound’s toxic dose (lethal dose at which 50% of test participants die after exposure to the molecule (LD_50_, mg/kg), toxicity class (class I–VI), toxicity targets and toxicological pathways using various computational models. All of the compounds were ranked in predicted toxicity class 4 with a LD_50_ prediction of 1000 mg/kg. The compounds containing a halogen substitute exhibited no active toxicity against any of the in silico toxicity target prediction models. The compounds containing nitro substituent exhibited active toxicity against both carcinogenicity and mutagenicity in silico target prediction models (Table S19). The SwissADME web-based server was employed tp predict the absorption, distribution, metabolism and exceretion (ADME) properties of the compounds (Figures S1–S9). The compounds passed four of the five criteria of the Lipinski rule of five indicating that the compounds are predicted to be well absorbed and distributed. The halogen substituted compounds were predicted to have high gastrointestinal (GI) absorption whereas the nitro derivative was predicted to have low GI absorption. Only compound **2** was predicted to not be a P-glycoprotein (P-gp) substrate and none of the compounds were predicted to not permeate across the blood-brain barrier (BBB). However, previous studies have shown that the adamantane structure has been employed as a lipophilic carrier to transport the molecules into the central nervous system (CNS) and therefore CNS side-effects should be considered [58].

## 3. Materials and Methods

### 3.1. X-ray Crystallography and Theoretical Computations

The X-ray intensity data were measured at room temperature, 293–309 K, using Cu*K*α radiation (λ = 1.54184 Å) in an Xcalibur, Ruby, Gemini diffractometer equipped with a CCD plate detector (Agilent Technologies, Inc., Santa Clara, CA, USA). The collected frames were integrated with the CrysAlis PRO software package. Data were corrected for the absorption effect using the CrysAlis PRO software package by the empirical absorption correction using spherical harmonics, implemented in the SCALE3 ABSPACK scaling algorithm [59]. The structures were solved using SHELXT small molecule structure solution program [60], then completed by difference Fourier map, and refined using SHELX2014 [61]. The final anisotropic full-matrix least-squares refinements on F^2^ with 290, 272, and 266 variables converged at R1 = 6.2%, 5.1%, and 5.7% for the observed data, and R2 = 14.6%, 10.6% and 11.9% for all data, respectively. All the non-hydrogen atoms were refined anisotropically, while the hydrogen atoms were generated geometrically, placed in calculated positions (C−H = 0.93–0.98 Å), and included as riding contributions with isotropic displacement parameters set at 1.2–1.5 times the U_eq_ value of the parent atom. Molecular and supramolecular graphics were carried out using the Mercury software [62]. In order to obtain a better understanding of the crystal packing, the crystallographic analysis was complemented with theoretical calculations using the Crystallographic Information File (CIF) obtained from the X-ray results. Hirshfeld surfaces (HFs) [63] mapped over d_norm_ were calculated using TONTO computational system [64], a Fortran-based object-oriented system for quantum chemistry and crystallography, by the Becke’s three-parameter hybrid function with the non-local correlation of Lee-Yang-Parr (B3LYP) method at 6-31G(d,p) basis set [65,66]. Pairwise interaction energies and the corresponding energy frameworks were calculated using the CE-B3LYP energy model based on B3LYP/6-31G(d,p) quantum mechanical charge distribution for unperturbed monomers. In these calculations, the total interaction energy was modeled as the sum of the electrostatic (E_ele_), polarization (E_pol_), dispersion (E_dis_), and exchange-repulsion (E_rep_) terms based on molecular wavefunctions calculated applying the crystal symmetry obtained from X-ray crystallographic results [67,68]. The energy levels of the electron-donor HOMO (highest occupied molecular orbital) and electron-acceptor LUMO (lowest unoccupied molecular orbital) were calculated by the B3LYP method using the 6-31G (d,p) basis set using the CIF files obtained from the X-ray diffraction measurements. These models are implemented in *CrystalExplorer* 17 [69].

### 3.2. PASS and Molecular Docking Studies

#### 3.2.1. PASS Evaluation

The online PASS tool (http://www.way2drug.com/passonline/index.php, accessed on 1 December 2020) was used to predict potential biological activities of test compounds **1**–**3** [38]. All compounds were drawn as .mol files and loaded into the PASS platform. The complete raw data tables containing all the predictions are included in the supplementary file (Tables S20–S22). The number of activities in the PASS data set for each activity record can be found at: http://www.way2drug.com/passonline/index.php (accessed on 1 December 2020).

#### 3.2.2. Preparation of Protein and Ligands

To determine the most appropriate 11β-HSD1 holo X-ray structure for docking analysis two molecular docking approaches, namely self-docking and cross-docking, were employed. Sixteen holo 11β-HSD1 X-ray structures (PDB ID: *2RBE*, *3H6K*, *3HFG*, *3PDJ*, *3TFQ*, *4BB5*, *4C7J*, *4C7K*, *4XH5*, *4IJU*, *4IJV*, *4IJW*, *4K1L*, *5PGU*, *5PGY*, *5QII*) were retrieved from the protein data bank (PDB, https://www.rcsb.org, accessed on 26 July 2021) [44]. X-ray structures with a resolution < 2.5 Å and R-values < 2.8 were deemed to be acceptable for molecular docking. The appropriate chains of the X-ray homodimer structures were selected using PDB residue-property plots and native ligand interactions. The protein chains with the least number of outliers on the residue property plots and best interactions were chosen for docking. The protein structures were prepared and The Molecular Operating Environment (MOE) 2020 software suite [53] was used for docking studies with the following protocol: The unselected protein chains and their respective co-crystallized ligands, solvent and co-factors were removed. Thereafter, the water molecules further than 4.5 Å from the ligand were removed. Atoms further than 8 Å from the ligand were fixed and the receptor residues were tethered with a constraint value of 0.25 Å. The tethering of the protein residue heavy atoms within 8 Å of the ligand ensures that no artificial movements from the original coordinates will occur during energy minimization [70]. The proteins were structurally prepared and protonated through the utilization of the built-in MOE structure preparation and Protonate3D software tools using the default parameters. Finally, partial charges were corrected and energy minimization was conducted utilizing the following parameters; forcefield: MMFF94x, solvation: Born and gradient: 0.01. Once the structures were optimized the fixed and tethered constraints were removed for molecular docking. The docking algorithm, which was chosen for these experiments, was based on induced fit docking to allow for flexible interactions of the test ligand with the protein. Hence, the constraints were removed to ensure the active site side chains were flexible during induced fit docking. The prepared protein structures were saved in .moe file format. The ligands used for molecular docking were drawn using the ACD/ChemSketch [71] package and saved in .mol2 file format. Protonation and energy minimization of the ligands was conducted using the enzyme preparation parameters.

#### 3.2.3. Self-Docking Protocol

Self-docking is a molecular docking approach in which the native ligand of a protein structure is re-docked within the active site. Self-docking is used at first to validate the docking protocol as well as to determine the protein structure’s suitability to successfully dock the native ligand. Each ligand was docked using the docking parameters; placement: triangle matcher, placement score algorithm: London dG, returned poses: 30, refinement: induced fit, iterations: 1000, refinement score algorithm: GBVI/WSA dG, scored poses: 5. The successfulness of the docked ligands was determined using a RMSD-based criteria between the docked ligand poses and crystallographic poses. The RMSD based criteria: Top docked pose (lowest binding affinity score pose) with a RMSD below 2 Å, three poses or more with a RMSD less than 2 Å, lowest RMSD value of docked poses below 2 Å and average RMSD of all docked poses below 3 Å. Protein structures whose re-docked native ligands did not meet the above criteria requirements were rejected. The validated protein structures were used for cross-docking.

#### 3.2.4. Cross-Docking Protocol

Cross-docking is a molecular docking approach where non-native ligands are docked within X-ray structures of the same protein. The protein structure with the best overall results in the self-docking approach was used to align and superpose each protein structure. The average scores of the RMSD based criteria from the self-docking method was used to assess the success of the cross-docking results. The ligands were imported into a combined database and docked using the docking parameters; placement: triangle matcher, placement score algorithm: London dG, returned poses: 100, refinement: induced fit, iterations: 1000, refinement score algorithm: GBVI/WSA dG, scored poses: 5.

#### 3.2.5. Molecular Docking of Compounds **1**–**3** and CADD Designed Compounds

The ligands were imported into a combined database and docked using the cross-dock docking protocol. The best docked ligand conformation of each compound was selected using the following criteria: lowest binding affinity score within the top 5 binding conformations and best interactions with important 11β-HSD1 active site residues. The best binding pose of each compound was visually inspected and the interactions with the binding pocket residues were analyzed using the online server Protein-Ligand Interaction Profiler (PLIP, https://plip-tool.biotec.tu-dresden.de, accessed on 26 July 2021) [48], Pymol molecular graphics system [54] and (MOE) 2020 software suite [54]. The build-in scoring function of MOE, S-score, was used to predict the binding affinity (kcal/mol) of each ligand with the enzyme protein active site after docking.

### 3.3. In Silico Toxicity and ADME Prediction Property Prediction Studies

ProTox-II web-based toxicity prediction tool was employed to predict the toxic dose (mg/kg), toxicity class, toxicity targets and pathway of the compounds. The ProTox-II web-server can be accessed at: https://tox-new.charite.de/protox II/ [56] (accessed on 26 July 2021). SwissADME online ADME tool was employed to determine the ADME properties of the compounds. SwissADME can be accessed at: http://www.swissadme.ch [57] (accessed on 26 July 2021).

## 4. Conclusions

The crystal structures of compounds **1**, **2** and **3** were analyzed. While **2** crystallizes in the triclinic *P*-1 space group, **1** and **3** crystallize in the same monoclinic *P*2_1_/*c* space group. Since the only difference between them is the *para*-substitution in the aryl group, the electronic nature of these NO_2_ and halogen groups seems to have no influence over the formation of the solid. In fact, compounds **1** and **3** not only share the same space group but also, the intermolecular interactions have the same architecture. This behavior allows imagining that the volume of the substituents plays an important role in the form in which the supramolecular structure is built. Nevertheless, the pairwise interaction energies show that differences exist between the intermolecular forces, which influence the total energy packing. Instead, the HOMO and LUMO energies are influenced by the electron withdrawing characteristics of the NO_2_ and F/Cl groups modifying their reactivity. A decrease in the LUMO energy of compound **1** compared with **2** and **3**, accompanied by also a low value of the band gap characterizes this molecule as soft and the best electron acceptor. Self-docking as well as cross-docking protocols were conducted and holo X-ray structure *4C7J* was able to reproduce the native and non-native ligands crystallographic poses. Compounds **1**–**3** exhibited similar binding affinity scores compared to the co-crystallized ligand **4YQ**. However, the phenyl substitution conjugated to the triazole moiety shifted the position of the compounds towards the entrance of the 11β-HSD1 active site. The triazole moiety was also rotated out of plane away from the two important binding site residues (Ser 170 and Tyr 183). Based on these findings compounds **D1**–**D9** were proposed and docked into the 11β-HSD1. **D1**–**D9** contained an additional carbon linker between the phenyl substituent and triazole moiety. The positions of the nitro- and halogen substitutions on the benzylsulfanyl moiety were altered as well. When compared to compounds **1**–**3**, the majority of the series **D** compounds were orientated deeper within the 11β-HSD1 hydrophobic pocket and hydrogen bond binding interactions with important catalytic active site residues Ser 170 and Tyr 183 were formed. The halogen substituted compounds were predicted to have a high GI absorption, pass four of the 5 Lipinski rule of five criteria as well as exhibit no toxicity on various computational toxicity models and should be considered for further development.

## Figures and Tables

**Table 1:** Crystallographic data and structure refinement parameters of compounds **1**, **2** and **3**.

	Compound 1	Compound 2	Compound 3
**Crystal Data**			
CCDC number	2057900	2057902	2057904
Empirical formula	C_25_H_26_N_4_O_2_S	C_25_H_26_FN_3_S	C_25_H_26_ClN_3_S
Formula weight	446.56	419.56	436.01
Temperature (K)	309 (2)	293 (2)	293 (2)
Crystal system	Monoclinic	Triclinic	Monoclinic
Space group	*P*2_1_/*c*	*P*-1	*P*2_1_/*c*
*a, b, c* (Å)	22.2883 (10), 10.5839 (6), 9.3161 (5)	9.8006 (7), 10.0776 (8), 11.4789 (8)	22.0756 (6), 10.3022 (3), 9.6645 (3)
*α, β, γ* (°)	90, 92.888 (4), 90	80.671 (6), 75.836 (6), 78.137 (6)	90, 90.686 (2), 90
Volume (Å^3^)	2194.9 (2)	1068.29 (14)	2197.81 (11)
*Z*	4	2	4
Radiation type	Cu *K*α	Cu *K*α	Cu *K*α
Calculated density (g/cm^3^)	1.351	1.304	1.318
*µ* (mm^−1^)	1.555	1.545	2.547
**Data Collection**			
Diffractometer	Xcalibur, Ruby, Gemini		
Absorption correction	*Multi-scan (CrysAlis PRO; Agilent, 2015)*		
*T_min_, T_max_*	0.830, 0.925	0.846, 0.884	0.760, 0.822
No. of measured, independent and observed [*I* > 2σ(*I*)] reflections	21797, 4556, 2065	20820, 4448, 2577	21912, 4579, 2423
*R*_int_	0.11	0.083	0.093
Theta range for data collection	−27 ≤ h ≤ 27, −13 ≤ k ≤ 13, −11 ≤ l ≤ 10	−12 ≤ h ≤ 12, −12 ≤ k ≤ 12, −14 ≤ l ≤ 12	−27 ≤ h ≤ 27, −12 ≤ k ≤ 12, −11 ≤ l ≤ 12
**Refinement**			
*R*[*F*^2^ > 2σ(*F*^2^)], *wR*(*F*^2^), *S*	0.062, 0.172, 0.99	0.051, 0.143, 1.00	0.057, 0.153, 1.04
No. of reflections	4508	4388	4512
H-atom treatment	H-atom parameters constrained		
Δρ_max_, Δρ_min_ (e Å^−3^)	0.222, −0.277	0.18, −0.19	0.21, −0.29
RMS deviation	0.047	0.042	0.043

**Table 2:** Selected hydrogen-bond geometry (Å, °) for compound **1**, **2** and **3**.

D‒H···A	D‒H	H···A	D···A	D‒H···A
**Compound 1**				
C4‒H4···N1^i^	0.93	2.59	3.327(5)	136
C14‒H14B···N2^ii^	0.97	2.61	3.529(5)	158
C19‒H19A···O1^iii^	0.97	2.63	3.470(5)	145
π···π^iii^ (Cg1 *^a^*···Cg1 *^a^*)	---	---	3.815(2) *	---
C22‒H22···O2^iv^	0.93	2.69	3.425(5)	140
**Compound 2**				
C24‒H24···N1^v^	0.93	2.56	3.465(4)	164
C4‒H4···F1^vi^	0.93	2.64	3.249(4)	124
C5‒H5···F1^vi^	0.93	2.64	3.249(4)	124
C15‒H15B···Cg2 *^b^*^,vii^	0.97	2.95	3.812(3)	149
C12‒H12A···Cg1 *^a^*^,viii^	0.97	2.84	3.683(3)	146
**Compound 3**				
C4‒H4···N1^i^	0.93	2.49	3.248(4)	139
C14‒H14A···N2^ii^	0.97	2.66	3.581(4)	160
C19‒H19B···Cl1^iii^	0.97	2.86	3.794(4)	161
π···π^iii^ (Cg1 *^a^*···Cg1 *^a^*)	---	---	3.931(3) *	---

Symmetry codes: (i) x, 1/2 − y, −1/2 + z; (ii) x, 1/2 − y, 1/2 + z; (iii) 1 − x, −y, 1 − z; (iv) 1 − x, −1/2 + y, 3/2 − z; (v) −x, 1 − y, 1 − z; (vi) 1 + x, −1 + y, z; (vii) 1 − x, −y, 1 − z; * Distance between centroids without hydrogen bridge. *^a^* Cg1 is the centroid of the C20/C25 ring. *^b^* Cg2 is the centroid of the C1/N1 ring.

**Table 3:** Selected CrystalExplorer CE-B3LYP interaction energies (kJ/mol) for compounds **1**, **2** and **3**. N is the number of molecules with a molecular centroid-to-centroid distance R (Å). Electron density was calculated using B3LYP/6-31G(d,p) model energies. Symop is the symmetry operation. Note: scale factors used to determine E_tot_: E_ele_ = 1.057; E_po_l = 0.740; E_dis_ = 0.871; E_rep_ = 0.618.

N	Symop	R	E_ele_	E_pol_	E_dis_	E_rep_	E_tot_
**Compound 1**							
2	x, −y + 1/2, z + 1/2	5.09	−25.9	−11.6	−93.9	69.7	−74.7
1	−x, −y, −z	12.53	−13.9	−2.3	−40.8	22.6	−38.1
2	−x, y + 1/2, −z + 1/2	12.02	−14.3	−3.7	−14.2	8.8	−24.8
2	x, y, z	10.58	2.3	−0.9	−9.0	2.4	−4.6
1	−x, −y, −z	15.06	2.2	−1.1	−4.2	2.1	−0.8
2	x, y, z	9.32	1.3	−0.5	−8.2	0.8	−5.7
2	x, −y + 1/2, z + 1/2	9.73	−1.0	−1.0	−37.1	16.4	−24.0
1	−x, −y, −z	10.67	−9.6	−3.0	−12.9	6.9	−19.4
1	−x, −y, −z	12.73	−2.7	−0.3	−22.6	9.6	−16.8
2	−x, y + 1/2, -z + 1/2	13.50	−0.5	−0.3	−11.9	3.4	−9.0
1	−x, −y, −z	14.52	−3.5	−0.1	−20.7	12.6	−14.1
**Compound 2**							
2	x, y, z	9.80	−4.4	−1.5	−40.2	21.6	−27.4
1	−x, −y, −z	8.99	−15.9	−7.5	−27.7	25.6	−30.6
2	x, y, z	12.53	−4.2	−0.9	−8.9	5.3	−9.6
1	−x, −y, −z	5.63	−15.3	−5.8	−101.8	53.3	−76.2
1	−x, −y, −z	10.21	−10.9	−1.7	−33.3	22.3	−28.1
2	x, y, z	13.14	−1.1	−0.2	−14.7	7.0	−9.8
1	−x, −y, −z	7.04	−14.2	−4.2	−59.8	32.4	−50.2
1	−x, −y, −z	12.33	−0.4	−0.2	−16.0	4.2	−12.0
1	−x, −y, −z	9.51	−9.7	−1.7	−27.6	22.0	−22.0
1	−x, −y, −z	11.19	−1.6	−0.1	−4.3	0.2	−5.4
2	x, y, z	11.48	−0.2	−0.4	−8.1	2.4	−6.1
1	−x, −y, −z	10.49	0.3	−0.3	−3.5	0.1	−2.9
1	−x, −y, −z	13.22	−0.0	−0.0	−2.7	0.0	−2.4
**Compound 3**							
1	−x, −y, −z	14.56	−0.4	−0.3	−6.9	5.2	−3.5
2	x, −y + 1/2, z + 1/2	5.24	−29.8	−11.5	−84.8	67.1	−72.4
1	−x, −y, −z	12.67	−11.0	−1.5	−32.3	25.5	−25.2
2	x, y, z	10.30	0.1	−0.8	−10.3	2.9	−7.7
2	−x, y + 1/2, −z + 1/2	11.74	−2.1	−0.7	−14.0	7.3	−10.5
2	x, y, z	9.66	1.1	−0.3	−6.3	0.3	−4.4
2	x, −y + 1/2, z + 1/2	9.58	−2.5	−0.8	−37.5	17.6	−25.0
1	−x, −y, −z	14.51	−2.9	−0.1	−18.0	10.5	−12.4
1	−x, −y, −z	10.90	−1.8	−0.6	−9.2	1.9	−9.1
1	−x, −y, −z	12.41	−2.5	−0.2	−21.6	8.3	−16.5
2	−x, y + 1/2, −z + 1/2	13.26	−1.0	−0.3	−14.2	5.8	−10.1

**Table 4:** HOMO and LUMO orbital energies (eV) and global reactivity descriptors (eV) for compounds **1**, **2** and **3**.

Parameters	Compound 1	Compound 2	Compound 3
HOMO energy	−6.27	−5.98	−6.06
LUMO energy	−2.27	−0.80	−0.81
HOMO–LUMO energy gap	4.00	5.18	5.25
Ionization Potential (IP)	6.27	5.98	6.06
Electron Affinity (EA)	2.27	0.80	0.81
Chemical Potential (*μ*)	−4.27	−3.39	−3.44
LUMO energy	−2.27	−0.80	−0.81
HOMO–LUMO energy gap	4.00	5.18	5.25
Ionization Potential (IP)	6.27	5.98	6.06
Electron Affinity (EA)	2.27	0.80	0.81
Chemical Potential (*μ*)	−4.27	−3.39	−3.44

**Table 5:** Top 10 PASS predictions for the activity spectrum of compounds **1**, **2** and **3**.

Compound 1			Compound 2			Compound 3		
Pa ^1^	Pi ^2^	Activity	Pa ^1^	Pi ^2^	Activity	Pa ^1^	Pi ^2^	Activity
0.619	0.002	**11** **β-HSD inhibitor**	0.738	0.005	**Anti-obesity**	0.783	0.005	**Anti-obesity**
0.610	0.002	**11** **β-HSD inhibitor**	0.687	0.005	Atherosclerosis treatment	0.676	0.002	**11** **β-HSD inhibitor**
0.596	0.09	Atherosclerosis treatment	0.673	0.002	**11** **β-HSD inhibitor**	0.667	0.002	**11ß-HSD1 inhibitor**
0.573	0.026	Antiviral (Picornavirus)	0.665	0.002	**11** **β-HSD inhibitor**	0.665	0.005	Atherosclerosis treatment
0.531	0.017	**Anti-obesity**	0.563	0.016	**Anti-diabetic**	0.566	0.015	**Anti-diabetic**
0.510	0.006	Antiparkinsonian	0.488	0.011	**Anti-diabetic (type 2)**	0.504	0.006	Antiparkinsonian
0.503	0.022	Antiviral (Influenza)	0.515	0.088	Kidney function stimulant	0.482	0.011	**Anti-diabetic (type 2)**
0.518	0.085	Kidney function stimulant	0.405	0.020	Antiparkinsonian	0.488	0.056	Antiviral (Picornavirus)
0.425	0.037	**Anti-diabetic**	0.378	0.009	Calcium channel blocker	0.515	0.088	Kidney function stimulant
0.399	0.019	**Anti-diabetic (type 2)**	0.341	0.012	Specificity phosphatase inhibitor	0.471	0.060	**Insulysin inhibitor**

^1^ Pa represents probability to be active. ^2^ Pi represents probability to be inactive. Bolded activities are related to 11β-HSD inhibitory action.

**Table 6:** Self -docking results of the sixteen holo 11β-HSD1 X-ray structure using the docking evaluation criteria.

Enzyme (PDB ID)	Top Pose RMSD (Å)		Lowest RMSD Pose of Top 5 Poses ^a^		Averg. RMSD of Top 5 Poses (Å)		No. Poses with a RMSD < 2 Å	
2BRE	1.10		0.97 (2)		2.46		3	
3H6K	8.88		2.58 (2)		6.36		0	
3HFG	1.60		1.23 (2)		2.02		3	
3DPJ	1.45		1.45 (1)		7.41		1	
3TFQ	7.94		3.14 (5)		6.88		0	
4BB5	2.37		1.49 (4)		2.26		2	
4C7J	1.53		0.72 (4)		1.25 *		5 *	
4C7K	1.21		1.21 (1)		2.88		1	
4HX5	1.22		1.22 (1)		1.56		5 *	
4IJU	0.99		0.99 (1)		1.35		5 *	
4IJV	0.43 *		0.43 (1) *		2.72		4	
4IJW	0.60		0.60 (1)		1.53		4	
4K1L	2.92		1.36 (5)		1.89		3	
5PGU	1.29		1.02 (2)		3.27		3	
5PGY	5.61		1.93 (4)		4.62		1	
5QII	1.45		1.31 (3)		1.61		4	
RMSD color scale (Å)		≤1	≤2	≤3	≤4	≥4		
No. poses color scale		4	4	3	2	1	0	

* = Best result in reference to each docking evaluation criteria, ^a^ binding affinity score rank.

**Table 7:** The cross-docking results of the RMSD values of the native and non-native ligands with the lowest binding affinity scores. Columns represent the enzymes and rows represent the co-crystallized ligands.

PDB ID	*2RBE*	*3HFG*	*4C7J*	*4C7K*	*4HX5*	*4IJU*	*4IJV*	*4IJW*	*4K1L*	*5QII*
2RBE	**0.67**	2.31	2.55	2.50	1.01	2.50	2.08	1.78	1.88	5.21
3HFG	3.96	**9.13**	1.46	3.24	2.48	9.33	8.47	1.65	2.95	2.63
4C7J	3.35	7.85	**0.79**	1.31	2.20	2.57	7.94	7.71	3.86	3.69
4C7K	7.06	3.42	0.86	**3.21**	1.83	1.41	7.43	7.07	3.64	3.47
4HX5	13.24	12.79	6.46	2.70	**1.10**	1.70	6.81	6.51	5.87	6.44
4IJU	2.19	3.42	2.18	3.93	2.90	**1.81**	8.36	3.67	2.26	3.90
4IJV	3.77	1.68	1.25	3.63	0.84	8.44	**0.41**	1.66	1.09	1.50
4IJW	5.93	1.86	6.84	0.72	2.12	2.93	1.04	**1.54**	3.51	0.76
4K1L	5.72	3.02	5.94	3.17	1.59	3.11	2.18	1.69	**3.13**	1.93
5QII	2.82	1.00	0.88	0.96	2.39	2.97	1.04	0.82	3.61	**0.79**
Average	4.87	4.65	2.92	2.54	1.85	3.68	4.58	3.41	3.18	3.03
RMSD color scale (Å)	≤1		≤2		≤3		≤4		≥4	

Bold figures = Self-docking of native ligand into respective native enzyme.

## Data Availability

CIF files containing complete crystallographic information of compounds **1**, **2** and **3** were deposited at Cambridge Crystallographic Data Centre (CCDC), deposition numbers 2057900 (compound **1**), 2057902 (compound **2**) and 2057904 (compound **3**) are freely available at www.ccdc.cam.ac.uk/data_request/cif (accessed on 26 July 2021).

## Supplementary Materials

The following are available online, Table S1: The cross-docking results of the number of RMSD values of the native and non-native ligands ≤ 2Å, Table S2: The cross-docking results with the lowest RMSD values of the native and non-native ligands, Columns represent the enzymes and rows represent the co-crystallized ligands, Table S3: The cross-docking results of the average RMSD values of the native and non-native ligands, Table S4: The cross-docking binding affinity score results of native and non-native ligands. Rows represent the enzymes and columns represent the co-crystallized ligands, Table S5: Tabulated binding affinity scores of **4YQ**, compounds **1**–**3** and series **D** obtained from the built-in scoring function of MOE, S-score, Tables S6–S18: Tabulated and visual representations of binding interactions of **4YQ**, compounds **1**–**3** and compounds **D1**–**D9** within *4C7J* active site using PLIP and Pymol molecular graphics system. Table S19: Tabulated toxicity prediction results of compounds **1**–**3** and **D1**–**D9** obtained from the web-based prediction tool Pro-Tox-II, Figures S1–S12: Visual representation of the predicted ADME results of compounds **1**–**3** and **D1**–**D9** obtained from the online ADME prediction tool SwissADME. Tables S20–S22: Complete data list of PASS predictions for the activity spectrum of compounds **1**–**3**.

## Sample Availability

Samples of compounds **1**, **2** and **3** are available from the corresponding authors.
