# Supplementary material for: Structural Insights and Docking Analysis of Adamantane-Linked 1,2,4-Triazole Derivatives as Potential 11β-HSD1 Inhibitors
Source: Molecules. 2021 Sep 2;26(17):5335. doi: 10.3390/molecules26175335 (PMC8433897; doi:10.3390/molecules26175335)
Supplement: Supplementary file 1 [file molecules-26-05335-s001.zip › molecules-1335659-supplementary.pdf]

Supporting Information

Structural Insights and Docking Analysis of Adamantane-Linked 1,2,4-Triazole Derivatives as Potential 11β-HSD1 Inhibitors

Doaa A. Osman [1], Mario A. Macías [2], Lamya H. Al-Wahaibi [3,*], Nora H. Al-Shaalan [3], Luke S. Zondagh [4], Jacques Joubert [4], Santiago Garcia-Granda [1] and Ali A. El-Emam [5,*]

Table S1. The cross-docking results of the number of RMSD values of the native and non-native ligands ≤ 2Å. Columns represent the enzymes and rows represent the co-crystallized ligands.

PDB ID	*2RBE*	*3HFG*	*4C7J*	*4C7K*	*4HX5*	*4IJU*	*4IJV*	*4IJW*	*4K1L*	*5QII*
2RBE	**4**	0	0	1	1	0	1	3	1	0
3HFG	0	**2**	2	0	0	0	2	4	2	2
4C7J	0	0	**4**	4	0	0	0	0	0	0
4C7K	1	1	4	**3**	1	1	0	0	0	0
4HX5	0	0	0	0	**4**	2	0	0	0	0
4IJU	0	0	0	0	0	**5**	0	0	0	0
4IJV	0	4	1	0	3	0	**5**	2	1	1
4IJW	0	3	2	3	1	0	3	**5**	2	4
4K1L	1	2	2	1	2	1	2	4	**0**	5
5QII	0	5	1	4	3	0	2	5	1	**5**
Average	6	17	16	16	15	9	15	23	7	17

Bold figures = Self-docking of native ligand into respective native enzyme.

No. poses color scale (Å) 5 4 3 2 1 0

Table S2. The cross-docking results with the lowest RMSD values of the native and non-native ligands. Columns represent the enzymes and rows represent the co-crystallized ligands.

PDB ID	*2RBE*	*3HFG*	*4C7J*	*4C7K*	*4HX5*	*4IJU*	*4IJV*	*4IJW*	*4K1L*	*5QII*
2RBE	**0.39**	2.09	2.00	1.46	1.01	2.02	1.82	1.77	1.88	2.03
3HFG	3.56	**1.36**	1.27	3.19	2.48	3.90	1.60	1.30	1.47	1.83
4C7J	2.87	2.42	**0.79**	1.31	2.20	2.07	7.86	3.66	2.33	2.78
4C7K	1.45	1.68	0.86	**1.11**	1.83	1.41	3.30	3.03	3.34	3.27
4HX5	3.17	6.01	2.22	2.47	**1.00**	1.70	5.76	5.73	5.87	5.94
4IJU	2.07	2.46	2.18	2.01	2.90	**0.92**	3.65	3.59	2.14	3.70
4IJV	3.28	1.11	1.25	2.39	0.84	3.13	**0.41**	1.13	1.09	1.50
4IJW	3.19	0.82	0.48	0.72	1.01	2.81	0.88	**0.64**	1.57	0.87
4K1L	0.89	1.17	1.77	1.95	1.13	1.49	1.47	1.34	**2.08**	1.11
5QII	2.82	0.93	0.88	0.96	0.82	2.72	0.90	0.52	1.07	**0.79**
Average	2.37	2.00	1.37	1.76	1.52	2.22	2.77	2.28	2.28	2.38

Bold figures = Self-docking of native ligand into respective native enzyme.

No. poses color scale (Å) ≤ 1 ≤ 2 ≤ 3 ≤ 4 ≤ 5

Table S3. The cross-docking results of the average RMSD values of the native and non-native ligands. Columns represent the enzymes and rows represent the co-crystallized ligands.

PDB ID	*2RBE*	*3HFG*	*4C7J*	*4C7K*	*4HX5*	*4IJU*	*4IJV*	*4IJW*	*4K1L*	*5QII*
2RBE	1.80	3.04	4.48	2.86	4.29	4.42	4.39	1.95	3.64	5.21
3HFG	3.81	4.59	2.66	4.32	2.92	7.29	5.65	1.76	2.26	4.43
4C7J	3.13	5.73	1.31	1.72	4.84	2.35	8.03	4.53	3.52	4.25
4C7K	4.39	3.72	2.48	2.05	3.15	3.54	5.62	4.85	3.48	3.39
4HX5	9.48	10.13	3.17	2.64	1.39	2.17	7.69	6.04	6.22	6.38
4IJU	2.26	3.82	4.30	3.28	5.05	1.51	7.45	4.46	2.75	3.77
4IJV	5.28	1.56	4.68	5.38	2.84	7.21	0.98	1.97	5.07	6.56
4IJW	5.36	1.77	4.96	1.71	3.08	3.72	3.33	1.34	3.48	2.23
4K1L	4.26	3.02	3.46	3.04	2.76	3.25	2.82	1.70	2.94	1.40
5QII	3.52	1.29	5.81	1.48	1.55	3.71	4.58	1.93	2.54	0.96
Average	4.33	3.87	3.73	2.85	3.19	3.92	5.053	3.05	3.59	3.86

Bold figures = Self-docking of native ligand into respective native enzyme.

No. poses color scale (Å) | ≤ 1 | ≤ 2 | ≤ 3 | ≤ 4 | > 4 |

Table S4. The cross-docking binding affinity results of native and non-native ligands. Rows represent the enzymes and columns represent the co-crystallized ligands.

PDB ID	*2RBE*	*3HFG*	*4C7J*	*4C7K*	*4HX5*	*4IJU*	*4IJV*	*4IJW*	*4K1L*	*5QII*
2RBE	**- 6.56**	- 7.26	- 7.78	- 6.92	- 7.26	- 6.84	- 6.63	- 6.45	- 6.63	- 6.68
3HFG	- 6.01	**- 8.09**	- 7.91	- 7.55	- 8.59	- 7.25	- 7.99	- 6.70	- 6.56	- 7.22
4C7J	- 5.77	- 7.51	**- 8.37**	- 7.87	- 8.87	- 7.18	- 7.53	- 7.08	- 6.31	- 7.37
4C7K	- 6.28	- 7.53	- 8.61	**- 8.80**	- 9.25	- 7.63	- 7.32	- 7.26	- 6.66	- 7.67
4HX5	- 5.77	- 7.44	- 7.65	- 7.34	**- 9.85**	- 6.73	- 7.37	- 7.28	- 6.45	- 7.49
4IJU	- 6.24	- 8.12	- 8.19	- 8.32	- 8.71	**- 7.58**	- 7.88	- 7.09	- 6.69	- 7.42
4IJV	- 6.15	- 8.39	- 7.79	- 8.07	- 8.47	- 6.84	**- 8.32**	- 7.25	- 6.53	- 7.55
4IJW	- 5.91	- 8.01	- 7.51	- 7.79	- 8.16	- 7.00	- 7.79	**- 7.37**	- 6.66	- 7.96
4K1L	- 6.61	- 7.22	- 7.77	- 8.05	- 7.79	- 6.94	- 6.75	- 6.91	**- 7.05**	- 6.86
5QII	- 5.92	- 7.65	- 8.41	- 8.36	- 8.08	- 7.23	- 7.14	- 7.33	- 6.90	**- 7.30**

Table S5. Tabulated binding affinity scores of **4YQ**, compounds **1-3** and series **D** obtained from the built-in scoring function of MOE, S-score.

Compound	Binding affinity scores (S-score) (kcal/mol)
**4YQ**	- 8.20
**1**	- 8.30
**2**	- 7.70
**3**	- 7.83
**D1**	- 8.24
**D2**	- 8.29
**D3**	- 7.98
**D4**	- 8.08
**D5**	- 8.02
**D6**	- 8.48
**D7**	- 8.31
**D8**	- 8.19
**D9**	- 8.29

Table S6. Tabulated and visual representations of binding interactions of **4YQ** within *4C7J* active site using PLIP and Pymol molecular graphics system.

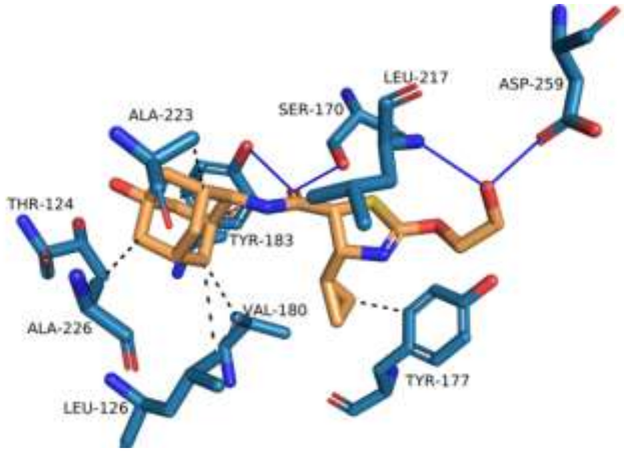

Residue	Amino acid	Distance (Å)	Type of interaction
121A	ILE	3.71	Hydrophobic
126A	LEU	3.89	Hydrophobic
177A	TYR	3.85	Hydrophobic
180A	VAL	3.86	Hydrophobic
183A	TYR	3.22	Hydrophobic
183A	TYR	3.60	Hydrophobic
223A	ALA	3.84	Hydrophobic
170A	SER	2.63	Hydrogen
183A	TYR	2.77	Hydrogen
217A	LEU	2.40	Hydrogen
259A	ASP	3.02	Hydrogen

Table S7. Tabulated and visual representations of binding interactions of compound **1** within *4C7J* active site using PLIP and Pymol molecular graphics system.

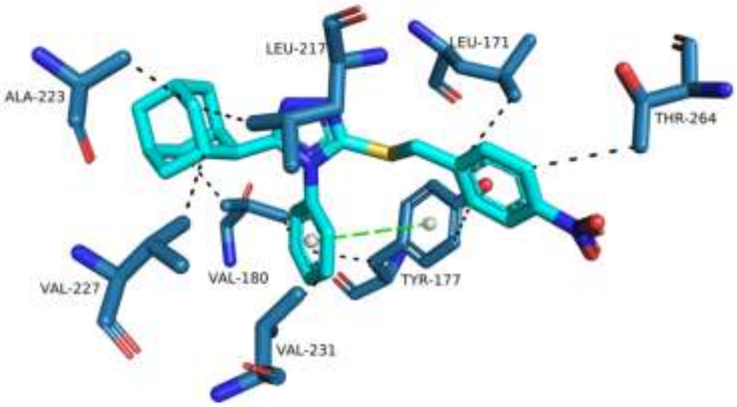

Residue	Amino acid	Distance (Å)	Type of interaction
171A	LEU	3.91	Hydrophobic
177A	TYR	3.71	Hydrophobic
177A	TYR	3.78	Hydrophobic
180A	VAL	3.55	Hydrophobic
180A	VAL	3.68	Hydrophobic
217A	LEU	3.49	Hydrophobic
223A	ALA	3.52	Hydrophobic
227A	VAL	3.76	Hydrophobic
231A	VAL	3.49	Hydrophobic
264A	THR	3.73	Hydrophobic
177A	TYR	4.76	π-π stacking

Table S8. Tabulated and visual representations of binding interactions of compound **2** within *4C7J* active site using PLIP and Pymol molecular graphics system.

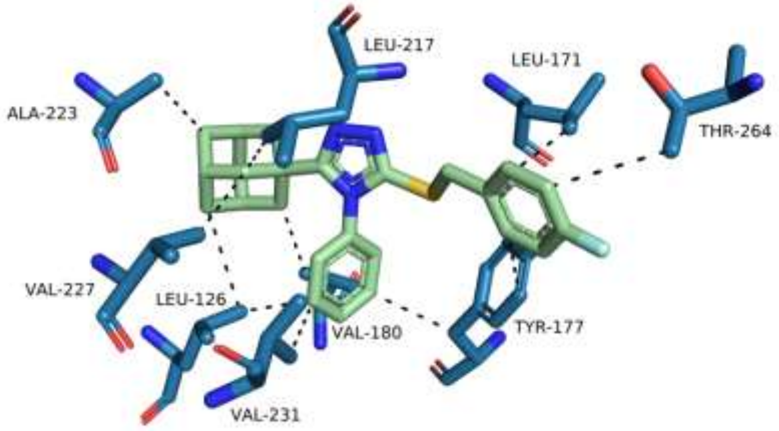

Residue	Amino acid	Distance (Å)	Type of interaction
126A	LEU	3.77	Hydrophobic
126A	LEU	3.13	Hydrophobic
171A	LEU	3.96	Hydrophobic
177A	TYR	3.48	Hydrophobic
177A	TYR	3.53	Hydrophobic
180A	VAL	3.67	Hydrophobic
180A	VAL	3.58	Hydrophobic
217A	LEU	3.40	Hydrophobic
223A	ALA	3.61	Hydrophobic
227A	VAL	3.84	Hydrophobic
231A	VAL	3.43	Hydrophobic
264A	THR	3.70	Hydrophobic

Table S9. Tabulated and visual representations of binding interactions of compound **3** within *4C7J* active site using PLIP and Pymol molecular graphics system.

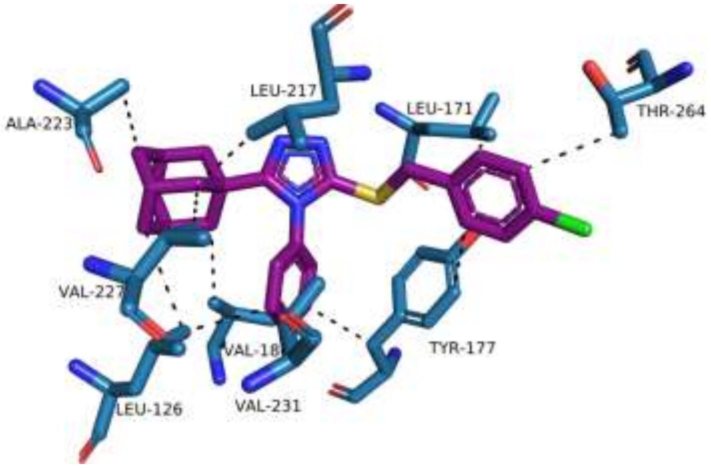

Residue	Amino acid	Distance (Å)	Type of interaction
126A	LEU	3.77	Hydrophobic
126A	LEU	3.14	Hydrophobic
171A	LEU	3.96	Hydrophobic
177A	TYR	3.46	Hydrophobic
177A	TYR	3.53	Hydrophobic
180A	VAL	3.65	Hydrophobic
180A	VAL	3.58	Hydrophobic
217A	LEU	3.40	Hydrophobic
223A	ALA	3.62	Hydrophobic
227A	VAL	3.86	Hydrophobic
231A	VAL	3.43	Hydrophobic
264A	THR	3.76	Hydrophobic

Table S10. Tabulated and visual representations of binding interactions of compound **D1** within *4C7J* active site using PLIP and Pymol molecular graphics system.

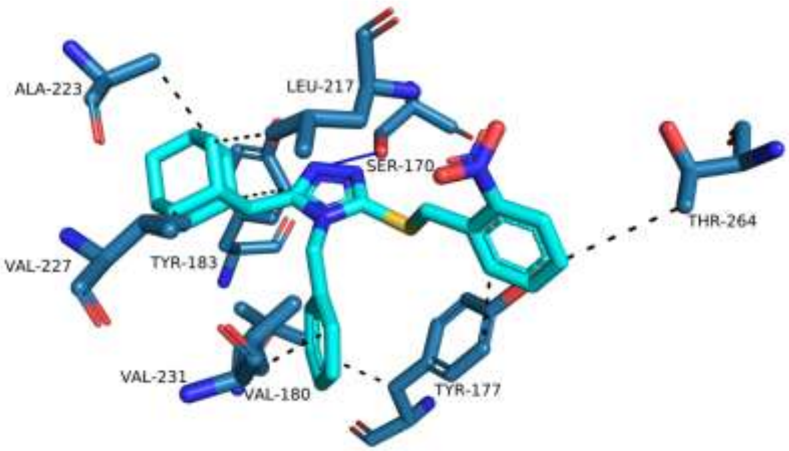

Residue	Amino acid	Distance (Å)	Type of interaction
177A	TYR	3.40	Hydrophobic
177A	TYR	3.48	Hydrophobic
180A	VAL	3.69	Hydrophobic
183A	TYR	3.98	Hydrophobic
217A	LEU	3.72	Hydrophobic
223A	ALA	3.44	Hydrophobic
227A	VAL	3.85	Hydrophobic
231A	VAL	3.27	Hydrophobic
264A	THR	3.90	Hydrophobic
170A	SER	3.00	Hydrogen

Table S11. Tabulated and visual representations of binding interactions of compound **D2** within *4C7J* active site using PLIP and Pymol molecular graphics system.

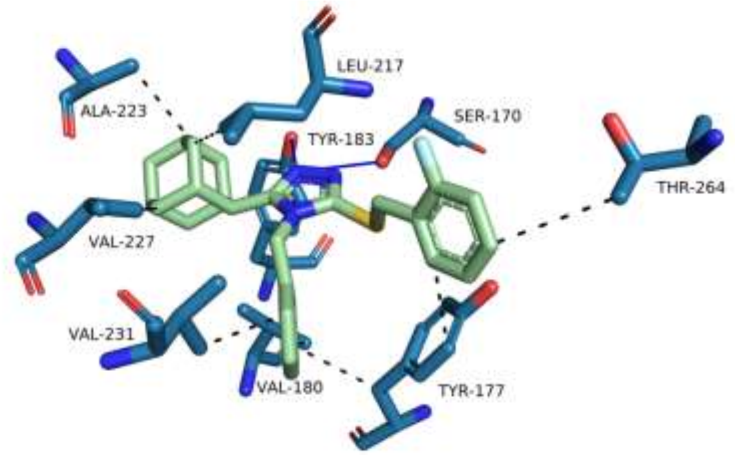

Residue	Amino acid	Distance (Å)	Type of interaction
177A	TYR	3.43	Hydrophobic
177A	TYR	3.46	Hydrophobic
180A	VAL	3.71	Hydrophobic
183A	TYR	3.99	Hydrophobic
217A	LEU	3.84	Hydrophobic
223A	ALA	3.40	Hydrophobic
227A	VAL	3.87	Hydrophobic
231A	VAL	3.30	Hydrophobic
264A	THR	3.92	Hydrophobic
170A	SER	2.93	Hydrogen
183A	TYR	3.01	Hydrogen

Table S12. Tabulated and visual representations of binding interactions of compound **D3** within *4C7J* active site using PLIP and Pymol molecular graphics system.

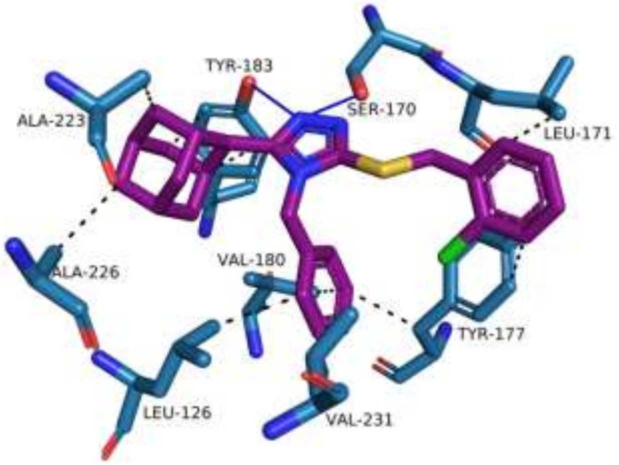

Residue	Amino acid	Distance (Å)	Type of interaction
126A	LEU	3.19	Hydrophobic
171A	LEU	3.29	Hydrophobic
177A	TYR	3.38	Hydrophobic
177A	TYR	3.47	Hydrophobic
180A	VAL	3.44	Hydrophobic
183A	TYR	3.63	Hydrophobic
183A	TYR	3.86	Hydrophobic
223A	ALA	3.74	Hydrophobic
226A	ALA	3.93	Hydrophobic
231A	VAL	3.45	Hydrophobic
170A	SER	2.54	Hydrogen
183A	TYR	2.42	Hydrogen

Table S13. Tabulated and visual representations of binding interactions of compound **D4** within *4C7J* active site using PLIP and Pymol molecular graphics system.

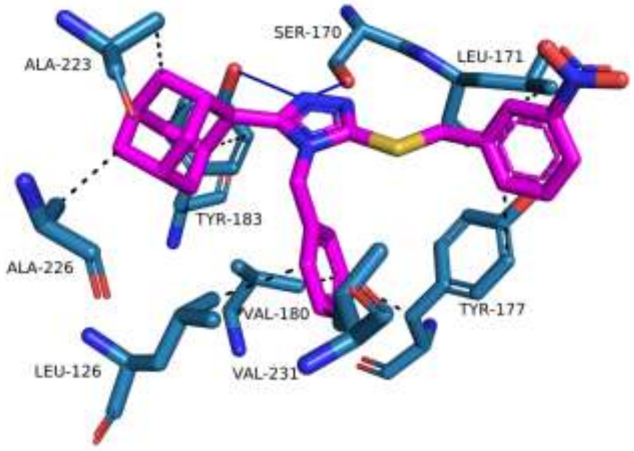

Residue	Amino acid	Distance (Å)	Type of interaction
126A	LEU	3.11	Hydrophobic
171A	LEU	3.58	Hydrophobic
177A	TYR	3.15	Hydrophobic
177A	TYR	3.44	Hydrophobic
180A	VAL	3.45	Hydrophobic
183A	TYR	3.63	Hydrophobic
183A	TYR	3.83	Hydrophobic
223A	ALA	3.71	Hydrophobic
226A	ALA	3.98	Hydrophobic
231A	VAL	3.34	Hydrophobic
170A	SER	2.45	Hydrogen
183A	TYR	2.63	Hydrogen

Table S14. Tabulated and visual representations of binding interactions of compound **D5** within *4C7J* active site using PLIP and Pymol molecular graphics system.

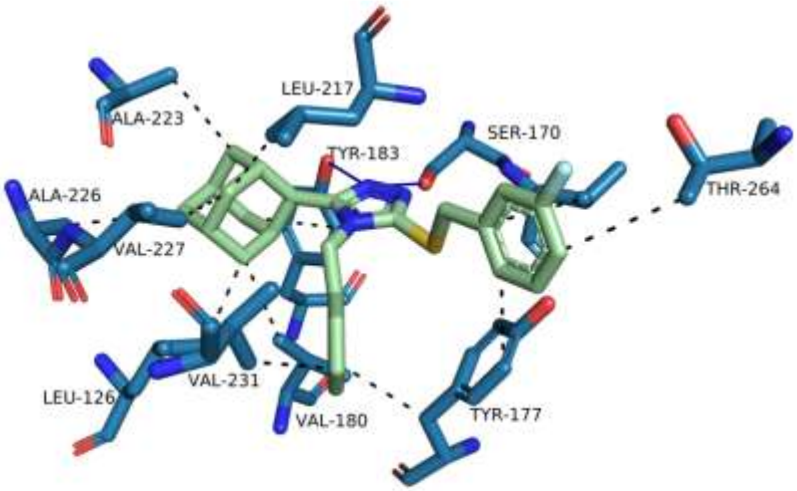

Residue	Amino acid	Distance (Å)	Type of interaction
126A	LEU	3.17	Hydrophobic
171A	LEU	3.95	Hydrophobic
177A	TYR	3.58	Hydrophobic
177A	TYR	3.56	Hydrophobic
180A	VAL	3.77	Hydrophobic
180A	VAL	3.67	Hydrophobic
183A	TYR	3.96	Hydrophobic
217A	LEU	3.40	Hydrophobic
223A	ALA	3.81	Hydrophobic
226A	ALA	3.95	Hydrophobic
227A	VAL	3.57	Hydrophobic
231A	VAL	3.34	Hydrophobic
264A	THR	3.77	Hydrophobic
170A	SER	2.34	Hydrogen
183A	TYR	3.10	Hydrogen

Table S15. Tabulated and visual representations of binding interactions of compound **D6** within *4C7J* active site using PLIP and Pymol molecular graphics system.

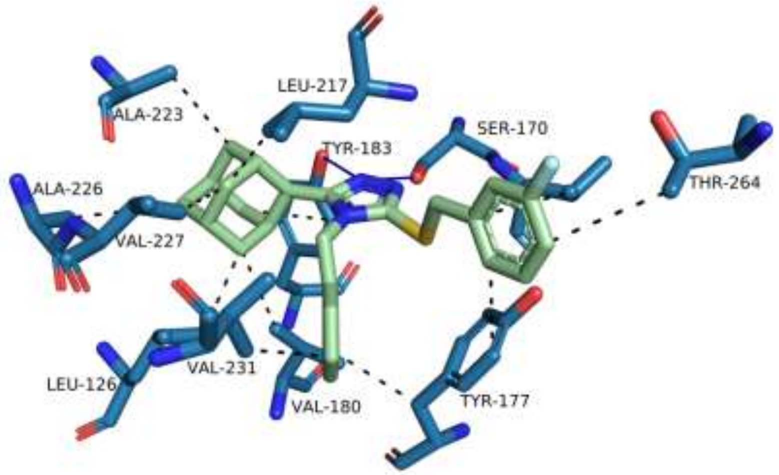

Residue	Amino acid	Distance (Å)	Type of interaction
126A	LEU	3.24	Hydrophobic
171A	LEU	3.66	Hydrophobic
177A	TYR	3.65	Hydrophobic
177A	TYR	3.44	Hydrophobic
180A	VAL	3.64	Hydrophobic
180A	VAL	3.64	Hydrophobic
183A	TYR	3.92	Hydrophobic
217A	LEU	3.74	Hydrophobic
223A	ALA	3.79	Hydrophobic
226A	ALA	3.96	Hydrophobic
227A	VAL	3.71	Hydrophobic
231A	VAL	3.26	Hydrophobic
264A	THR	3.83	Hydrophobic
170A	SER	2.52	Hydrogen

Table S16. Tabulated and visual representations of binding interactions of compound **D7** within *4C7J* active site using PLIP and Pymol molecular graphics system.

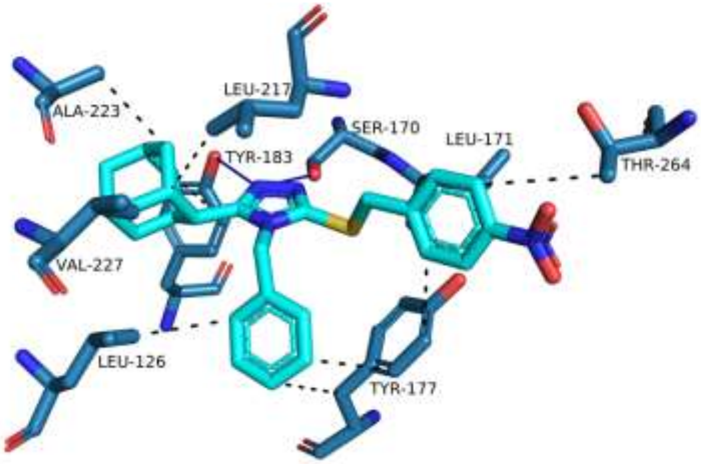

Residue	Amino acid	Distance (Å)	Type of interaction
126A	LEU	3.45	Hydrophobic
171A	LEU	3.93	Hydrophobic
177A	TYR	3.36	Hydrophobic
177A	TYR	3.60	Hydrophobic
177A	TYR	3.51	Hydrophobic
183A	TYR	3.85	Hydrophobic
217A	LEU	3.65	Hydrophobic
223A	ALA	3.62	Hydrophobic
227A	VAL	3.85	Hydrophobic
264A	THR	3.99	Hydrophobic
170A	SER	2.67	Hydrogen
183A	TYR	3.04	Hydrogen

Table S17. Tabulated and visual representations of binding interactions of compound **D8** within *4C7J* active site using PLIP and Pymol molecular graphics system.

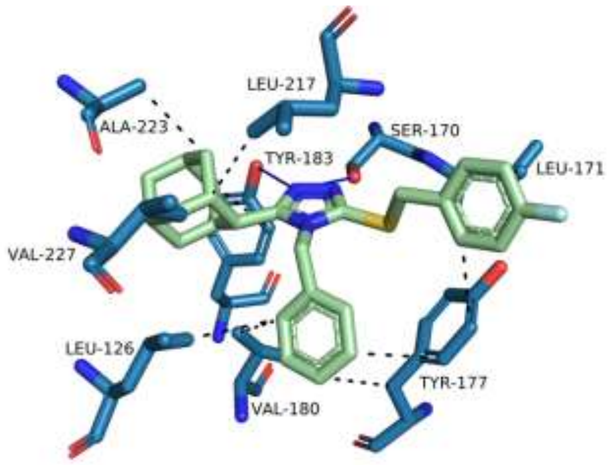

Residue	Amino acid	Distance (Å)	Type of interaction
126A	LEU	3.33	Hydrophobic
171A	LEU	3.90	Hydrophobic
177A	TYR	3.53	Hydrophobic
177A	TYR	3.67	Hydrophobic
177A	TYR	3.48	Hydrophobic
180A	VAL	3.78	Hydrophobic
183A	TYR	3.87	Hydrophobic
217A	LEU	3.74	Hydrophobic
223A	ALA	3.56	Hydrophobic
227A	VAL	3.91	Hydrophobic
170A	SER	2.72	Hydrogen
183A	TYR	2.97	Hydrogen

Table S18. Tabulated and visual representations of binding interactions of compound **D9** within *4C7J* active site using PLIP and Pymol molecular graphics system.

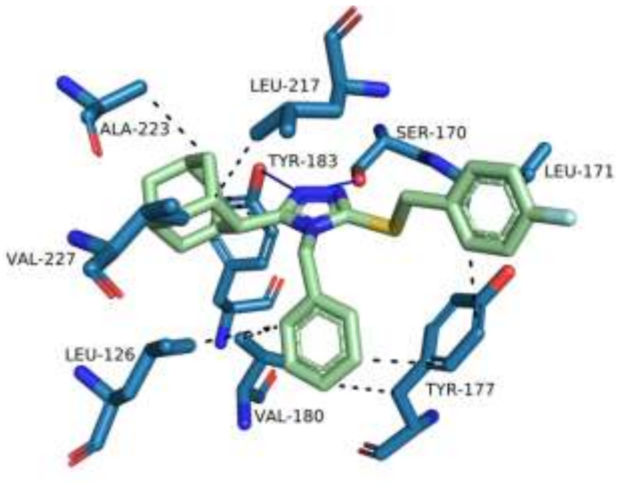

Residue	Amino acid	Distance (Å)	Type of interaction
126A	LEU	3.39	Hydrophobic
171A	LEU	3.92	Hydrophobic
177A	TYR	3.43	Hydrophobic
177A	TYR	3.62	Hydrophobic
177A	TYR	3.40	Hydrophobic
183A	TYR	3.86	Hydrophobic
217A	LEU	3.70	Hydrophobic
223A	ALA	3.57	Hydrophobic
227A	VAL	3.89	Hydrophobic
170A	SER	2.70	Hydrogen
183A	TYR	2.99	Hydrogen

Table S19. Tabulated toxicity prediction results of compounds 1-**3** and **D1-9** obtained from the web-based prediction tool ProTox-II

**Toxicity and target classification**	**Compounds**											
	**1**	**2**	**3**	**D1**	**D2**	**D3**	**D4**	**D5**	**D6**	**D7**	**D8**	**D9**
**Toxic Dose (mg/kg)**	1000	1000	1000	1000	1000	1000	1000	1000	1000	1000	1000	1000
**Toxicity class**	IV	IV	IV	IV	IV	IV	IV	IV	IV	IV	IV	IV
**Hepatotoxicity**	Yes	No	No	Yes	No	No	No	No	No	Yes	No	No
**Carcinogenicity**	Yes	No	No	Yes	No	No	No	No	No	Yes	No	No
**Immunitoxicity**	No	No	No	No	No	No	No	No	No	No	No	No
**Mutagenicity**	No	No	No	No	No	No	No	No	No	No	No	No
**Cytotoxicity**	No	No	No	No	No	No	No	No	No	No	No	No

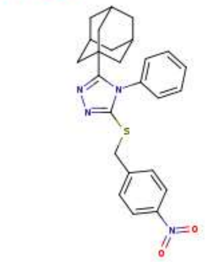

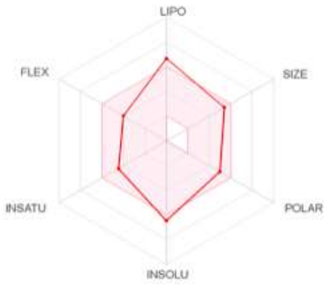

SMILES O=N(=O)c1ccc(cc1)CSc1nnc(n1c1ccccc1)C12CC3CC(C2)CC(C1)C3

Physicochemical Properties	
Formula	C25H26N4O2S
Molecular weight	446.56 g/mol
Num. heavy atoms	32
Num. arom. heavy atoms	17
Fraction Csp3	0.44
Num. rotatable bonds	6
Num. H-bond acceptors	4
Num. H-bond donors	0
Molar Refractivity	128.16
TPSA	101.83 Å²

Lipophilicity	
Log $P_{o/w}$ (iLOGP)	3.77
Log $P_{o/w}$ (XLOGP3)	6.14
Log $P_{o/w}$ (WLOGP)	6.31
Log $P_{o/w}$ (MLOGP)	5.35
Log $P_{o/w}$ (SILICOS-IT)	3.25
Consensus Log $P_{o/w}$	4.96

Water Solubility	
Log *S* (ESOL)	-6.47
Solubility	1.50e-04 mg/ml ; 3.36e-07 mol/l
Class	Poorly soluble
Log *S* (Ali)	-8.06
Solubility	3.88e-06 mg/ml ; 8.69e-09 mol/l
Class	Poorly soluble
Log *S* (SILICOS-IT)	-7.39
Solubility	1.82e-05 mg/ml ; 4.06e-08 mol/l
Class	Poorly soluble

Pharmacokinetics	
GI absorption	Low
BBB permeant	No
P-gp substrate	Yes
CYP1A2 inhibitor	No
CYP2C19 inhibitor	Yes
CYP2C9 inhibitor	Yes
CYP2D6 inhibitor	No
CYP3A4 inhibitor	Yes
Log K_p (skin permeation)	-4.66 cm/s

Druglikeness	
Lipinski	Yes; 1 violation: MLOGP>4.15
Ghose	No; 1 violation: WLOGP>5.6
Veber	Yes
Egan	No; 1 violation: WLOGP>5.88
Muegge	No; 1 violation: XLOGP3>5
Bioavailability Score	0.55

Medicinal Chemistry	
PAINS	0 alert
Brenk	1 alert: nitro_group
Leadlikeness	No; 2 violations: MW>350, XLOGP3>3.5
Synthetic accessibility	5.88

Figure S1: Visual representation of the predicted ADME results of compound **1** obtained from the online ADME prediction tool SwissADME.

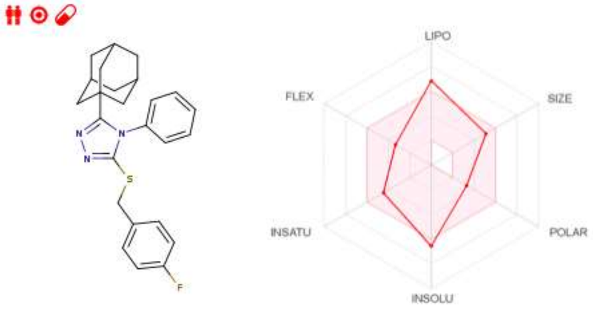

SMILES Fc1ccc(cc1)CSc1nnc(n1c1ccccc1)C12CC3CC(C2)CC(C1)C3

Physicochemical Properties	
Formula	C25H26FN3S
Molecular weight	419.56 g/mol
Num. heavy atoms	30
Num. arom. heavy atoms	17
Fraction Csp3	0.44
Num. rotatable bonds	5
Num. H-bond acceptors	3
Num. H-bond donors	0
Molar Refractivity	119.30
TPSA	56.01 Å²

Lipophilicity	
Log $P_{o/w}$ (iLOGP)	3.69
Log $P_{o/w}$ (XLOGP3)	6.41
Log $P_{o/w}$ (WLOGP)	6.43
Log $P_{o/w}$ (MLOGP)	6.00
Log $P_{o/w}$ (SILICOS-IT)	5.46
Consensus Log $P_{o/w}$	5.60

Water Solubility	
Log *S* (ESOL)	-6.57
Solubility	1.13e-04 mg/ml ; 2.70e-07 mol/l
Class	Poorly soluble
Log *S* (Ali)	-7.38
Solubility	1.75e-05 mg/ml ; 4.18e-08 mol/l
Class	Poorly soluble
Log *S* (SILICOS-IT)	-7.84
Solubility	6.12e-06 mg/ml ; 1.46e-08 mol/l
Class	Poorly soluble

Pharmacokinetics	
GI absorption	High
BBB permeant	No
P-gp substrate	No
CYP1A2 inhibitor	No
CYP2C19 inhibitor	No
CYP2C9 inhibitor	Yes
CYP2D6 inhibitor	Yes
CYP3A4 inhibitor	No
Log K_p (skin permeation)	-4.31 cm/s

Druglikeness	
Lipinski	Yes; 1 violation: MLOGP>4.15
Ghose	No; 1 violation: WLOGP>5.6
Veber	Yes
Egan	No; 1 violation: WLOGP>5.88
Muegge	No; 1 violation: XLOGP3>5
Bioavailability Score	0.55

Medicinal Chemistry	
PAINS	0 alert
Brenk	0 alert
Leadlikeness	No; 2 violations: MW>350, XLOGP3>3.5
Synthetic accessibility	5.76

Figure S2: Visual representation of the predicted ADME results of compound **2** obtained from the online ADME prediction tool SwissADME.

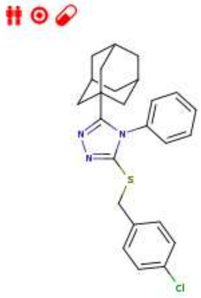

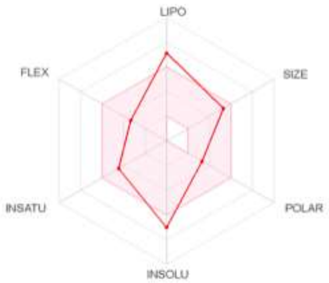

SMILES Clc1ccc(cc1)CSc1nnc(n1c1ccccc1)C12CC3CC(C2)CC(C1)C3

Physicochemical Properties	
Formula	C25H26ClN3S
Molecular weight	436.01 g/mol
Num. heavy atoms	30
Num. arom. heavy atoms	17
Fraction Csp3	0.44
Num. rotatable bonds	5
Num. H-bond acceptors	2
Num. H-bond donors	0
Molar Refractivity	124.35
TPSA	56.01 Å²

Lipophilicity	
Log $P_{o/w}$ (iLOGP)	3.81
Log $P_{o/w}$ (XLOGP3)	6.94
Log $P_{o/w}$ (WLOGP)	6.53
Log $P_{o/w}$ (MLOGP)	6.10
Log $P_{o/w}$ (SILICOS-IT)	5.68
Consensus Log $P_{o/w}$	5.81

Water Solubility	
Log *S* (ESOL)	-7.00
Solubility	4.31e-05 mg/ml ; 9.89e-08 mol/l
Class	Poorly soluble
Log *S* (Ali)	-7.93
Solubility	5.13e-06 mg/ml ; 1.18e-08 mol/l
Class	Poorly soluble
Log *S* (SILICOS-IT)	-8.16
Solubility	3.04e-06 mg/ml ; 6.97e-09 mol/l
Class	Poorly soluble

Pharmacokinetics	
GI absorption	High
BBB permeant	No
P-gp substrate	Yes
CYP1A2 inhibitor	No
CYP2C19 inhibitor	No
CYP2C9 inhibitor	Yes
CYP2D6 inhibitor	Yes
CYP3A4 inhibitor	No
Log K_p (skin permeation)	-4.03 cm/s

Druglikeness	
Lipinski	Yes; 1 violation: MLOGP>4.15
Ghose	No; 1 violation: WLOGP>5.6
Veber	Yes
Egan	No; 1 violation: WLOGP>5.88
Muegge	No; 1 violation: XLOGP3>5
Bioavailability Score	0.55

Medicinal Chemistry	
PAINS	0 alert
Brenk	0 alert
Leadlikeness	No; 2 violations: MW>350, XLOGP3>3.5
Synthetic accessibility	5.73

Figure S3: Visual representation of the predicted ADME results of compound **3** obtained from the online ADME prediction tool SwissADME.

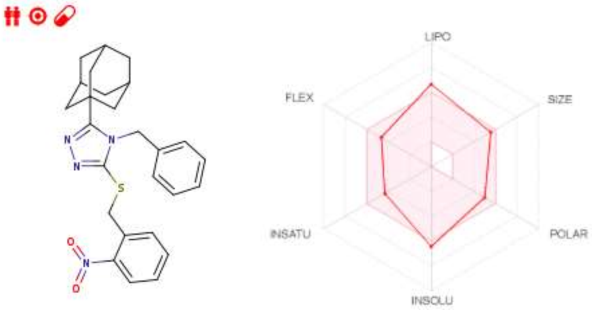

SMILES O=N(=O)c1ccccc1CSc1nnc(n1Cc1ccccc1)C12CC3CC(C2)CC(C1)C3

Physicochemical Properties	
Formula	C26H28N4O2S
Molecular weight	460.59 g/mol
Num. heavy atoms	33
Num. arom. heavy atoms	17
Fraction Csp3	0.46
Num. rotatable bonds	7
Num. H-bond acceptors	4
Num. H-bond donors	0
Molar Refractivity	132.57
TPSA	101.83 Å²

Lipophilicity	
Log $P_{o/w}$ (iLOGP)	3.83
Log $P_{o/w}$ (XLOGP3)	6.08
Log $P_{o/w}$ (WLOGP)	6.37
Log $P_{o/w}$ (MLOGP)	5.29
Log $P_{o/w}$ (SILICOS-IT)	3.65
Consensus Log $P_{o/w}$	5.04

Water Solubility	
Log *S* (ESOL)	-6.45
Solubility	1.65e-04 mg/ml ; 3.59e-07 mol/l
Class	Poorly soluble
Log *S* (Ali)	-8.00
Solubility	4.62e-06 mg/ml ; 1.00e-08 mol/l
Class	Poorly soluble
Log *S* (SILICOS-IT)	-7.78
Solubility	7.63e-06 mg/ml ; 1.66e-08 mol/l
Class	Poorly soluble

Pharmacokinetics	
GI absorption	Low
BBB permeant	No
P-gp substrate	Yes
CYP1A2 inhibitor	No
CYP2C19 inhibitor	Yes
CYP2C9 inhibitor	Yes
CYP2D6 inhibitor	Yes
CYP3A4 inhibitor	Yes
Log K_p (skin permeation)	-4.79 cm/s

Druglikeness	
Lipinski	Yes; 1 violation: MLOGP>4.15
Ghose	No; 2 violations: WLOGP>5.6, MR>130
Veber	Yes
Egan	No; 1 violation: WLOGP>5.88
Muegge	No; 1 violation: XLOGP3>5
Bioavailability Score	0.55

Medicinal Chemistry	
PAINS	0 alert
Brenk	1 alert: nitro_group
Leadlikeness	No; 2 violations: MW>350, XLOGP3>3.5
Synthetic accessibility	6.00

Figure S4: Visual representation of the predicted ADME results of compound D**1** obtained from the online based ADME prediction tool SwissADME.

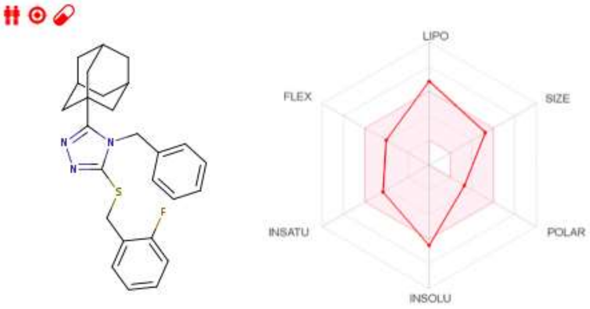

SMILES Fc1ccccc1CSc1nnc(n1Cc1ccccc1)C12CC3CC(C2)CC(C1)C3

Physicochemical Properties	
Formula	C26H28FN3S
Molecular weight	433.58 g/mol
Num. heavy atoms	31
Num. arom. heavy atoms	17
Fraction Csp3	0.46
Num. rotatable bonds	6
Num. H-bond acceptors	3
Num. H-bond donors	0
Molar Refractivity	123.71
TPSA	56.01 Å²

Lipophilicity	
Log $P_{o/w}$ (iLOGP)	3.69
Log $P_{o/w}$ (XLOGP3)	6.35
Log $P_{o/w}$ (WLOGP)	6.49
Log $P_{o/w}$ (MLOGP)	5.93
Log $P_{o/w}$ (SILICOS-IT)	5.85
Consensus Log $P_{o/w}$	5.66

Water Solubility	
Log *S* (ESOL)	-6.54
Solubility	1.25e-04 mg/ml ; 2.89e-07 mol/l
Class	Poorly soluble
Log *S* (Ali)	-7.32
Solubility	2.09e-05 mg/ml ; 4.82e-08 mol/l
Class	Poorly soluble
Log *S* (SILICOS-IT)	-8.23
Solubility	2.56e-06 mg/ml ; 5.92e-09 mol/l
Class	Poorly soluble

Pharmacokinetics	
GI absorption	High
BBB permeant	No
P-gp substrate	Yes
CYP1A2 inhibitor	No
CYP2C19 inhibitor	No
CYP2C9 inhibitor	Yes
CYP2D6 inhibitor	Yes
CYP3A4 inhibitor	Yes
Log K_p (skin permeation)	-4.44 cm/s

Druglikeness	
Lipinski	Yes; 1 violation: MLOGP>4.15
Ghose	No; 1 violation: WLOGP>5.6
Veber	Yes
Egan	No; 1 violation: WLOGP>5.88
Muegge	No; 1 violation: XLOGP3>5
Bioavailability Score	0.55

Medicinal Chemistry	
PAINS	0 alert
Brenk	0 alert
Leadlikeness	No; 2 violations: MW>350, XLOGP3>3.5
Synthetic accessibility	5.87

Figure S5: Visual representation of the predicted ADME results of compound **D2** obtained from the online ADME prediction tool SwissADME.

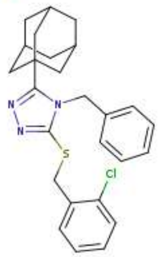

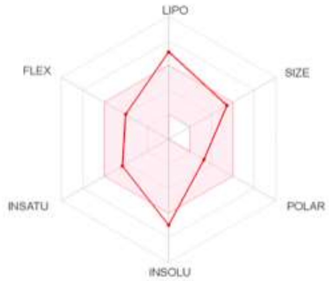

SMILES Clc1ccccc1CSc1nnc(n1Cc1ccccc1)C12CC3CC(C2)CC(C1)C3

Physicochemical Properties	
Formula	C26H28ClN3S
Molecular weight	450.04 g/mol
Num. heavy atoms	31
Num. arom. heavy atoms	17
Fraction Csp3	0.46
Num. rotatable bonds	6
Num. H-bond acceptors	2
Num. H-bond donors	0
Molar Refractivity	128.76
TPSA	56.01 Å²

Lipophilicity	
Log $P_{o/w}$ (iLOGP)	3.87
Log $P_{o/w}$ (XLOGP3)	6.87
Log $P_{o/w}$ (WLOGP)	6.59
Log $P_{o/w}$ (MLOGP)	6.03
Log $P_{o/w}$ (SILICOS-IT)	6.07
Consensus Log $P_{o/w}$	5.89

Water Solubility	
Log *S* (ESOL)	-6.97
Solubility	4.84e-05 mg/ml ; 1.08e-07 mol/l
Class	Poorly soluble
Log *S* (Ali)	-7.86
Solubility	6.26e-06 mg/ml ; 1.39e-08 mol/l
Class	Poorly soluble
Log *S* (SILICOS-IT)	-8.55
Solubility	1.27e-06 mg/ml ; 2.83e-09 mol/l
Class	Poorly soluble

Pharmacokinetics	
GI absorption	High
BBB permeant	No
P-gp substrate	Yes
CYP1A2 inhibitor	No
CYP2C19 inhibitor	No
CYP2C9 inhibitor	Yes
CYP2D6 inhibitor	Yes
CYP3A4 inhibitor	Yes
Log K_p (skin permeation)	-4.17 cm/s

Druglikeness	
Lipinski	Yes; 1 violation: MLOGP>4.15
Ghose	No; 1 violation: WLOGP>5.6
Veber	Yes
Egan	No; 1 violation: WLOGP>5.88
Muegge	No; 1 violation: XLOGP3>5
Bioavailability Score	0.55

Medicinal Chemistry	
PAINS	0 alert
Brenk	0 alert
Leadlikeness	No; 2 violations: MW>350, XLOGP3>3.5
Synthetic accessibility	5.86

Figure S6: Visual representation of the predicted ADME results of compound **D3** obtained from the online ADME prediction tool SwissADME.

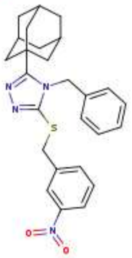

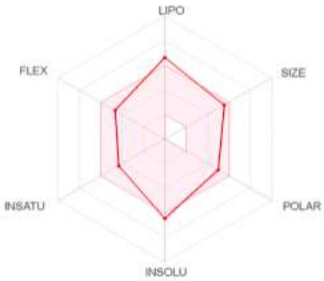

SMILES O=N(=O)c1cccc(c1)CSc1nnc(n1Cc1ccccc1)C12CC3CC(C2)CC(C1)C3

Physicochemical Properties	
Formula	C26H28N4O2S
Molecular weight	460.59 g/mol
Num. heavy atoms	33
Num. arom. heavy atoms	17
Fraction Csp3	0.46
Num. rotatable bonds	7
Num. H-bond acceptors	4
Num. H-bond donors	0
Molar Refractivity	132.57
TPSA	101.83 Å²

Lipophilicity	
Log $P_{o/w}$ (iLOGP)	3.78
Log $P_{o/w}$ (XLOGP3)	6.08
Log $P_{o/w}$ (WLOGP)	6.37
Log $P_{o/w}$ (MLOGP)	5.29
Log $P_{o/w}$ (SILICOS-IT)	3.65
Consensus Log $P_{o/w}$	5.03

Water Solubility	
Log *S* (ESOL)	-6.45
Solubility	1.65e-04 mg/ml ; 3.59e-07 mol/l
Class	Poorly soluble
Log *S* (Ali)	-8.00
Solubility	4.62e-06 mg/ml ; 1.00e-08 mol/l
Class	Poorly soluble
Log *S* (SILICOS-IT)	-7.78
Solubility	7.63e-06 mg/ml ; 1.66e-08 mol/l
Class	Poorly soluble

Pharmacokinetics	
GI absorption	Low
BBB permeant	No
P-gp substrate	Yes
CYP1A2 inhibitor	No
CYP2C19 inhibitor	Yes
CYP2C9 inhibitor	Yes
CYP2D6 inhibitor	Yes
CYP3A4 inhibitor	Yes
Log K_p (skin permeation)	-4.79 cm/s

Druglikeness	
Lipinski	Yes; 1 violation: MLOGP>4.15
Ghose	No; 2 violations: WLOGP>5.6, MR>130
Veber	Yes
Egan	No; 1 violation: WLOGP>5.88
Muegge	No; 1 violation: XLOGP3>5
Bioavailability Score	0.55

Medicinal Chemistry	
PAINS	0 alert
Brenk	1 alert: nitro_group
Leadlikeness	No; 2 violations: MW>350, XLOGP3>3.5
Synthetic accessibility	5.95

Figure S7: Visual representation of the predicted ADME results of compound D**4** obtained from the online ADME prediction tool SwissADME.

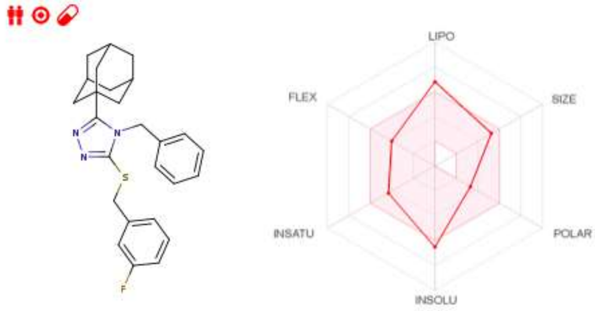

SMILES Fc1cccc(c1)CSc1nnc(n1Cc1ccccc1)C12CC3CC(C2)CC(C1)C3

Physicochemical Properties	
Formula	C26H28FN3S
Molecular weight	433.58 g/mol
Num. heavy atoms	31
Num. arom. heavy atoms	17
Fraction Csp3	0.46
Num. rotatable bonds	6
Num. H-bond acceptors	3
Num. H-bond donors	0
Molar Refractivity	123.71
TPSA	56.01 Å²

Lipophilicity	
Log $P_{o/w}$ (iLOGP)	3.61
Log $P_{o/w}$ (XLOGP3)	6.35
Log $P_{o/w}$ (WLOGP)	6.49
Log $P_{o/w}$ (MLOGP)	5.93
Log $P_{o/w}$ (SILICOS-IT)	5.85
Consensus Log $P_{o/w}$	5.65

Water Solubility	
Log *S* (ESOL)	-6.54
Solubility	1.25e-04 mg/ml ; 2.89e-07 mol/l
Class	Poorly soluble
Log *S* (Ali)	-7.32
Solubility	2.09e-05 mg/ml ; 4.82e-08 mol/l
Class	Poorly soluble
Log *S* (SILICOS-IT)	-8.23
Solubility	2.56e-06 mg/ml ; 5.92e-09 mol/l
Class	Poorly soluble

Pharmacokinetics	
GI absorption	High
BBB permeant	No
P-gp substrate	Yes
CYP1A2 inhibitor	No
CYP2C19 inhibitor	No
CYP2C9 inhibitor	Yes
CYP2D6 inhibitor	Yes
CYP3A4 inhibitor	Yes
Log K_p (skin permeation)	-4.44 cm/s

Druglikeness	
Lipinski	Yes; 1 violation: MLOGP>4.15
Ghose	No; 1 violation: WLOGP>5.6
Veber	Yes
Egan	No; 1 violation: WLOGP>5.88
Muegge	No; 1 violation: XLOGP3>5
Bioavailability Score	0.55

Medicinal Chemistry	
PAINS	0 alert
Brenk	0 alert
Leadlikeness	No; 2 violations: MW>350, XLOGP3>3.5
Synthetic accessibility	5.87

Figure S8: Visual representation of the predicted ADME results of compound **D5** obtained from the online ADME prediction tool SwissADME.

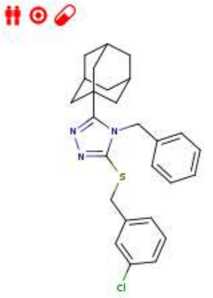

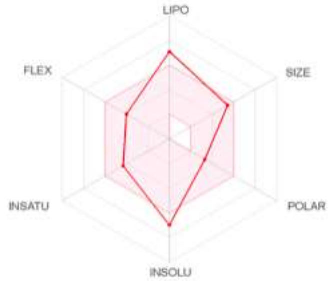

SMILES Clc1cccc(c1)CSc1nnc(n1Cc1ccccc1)C12CC3CC(C2)CC(C1)C3

Physicochemical Properties	
Formula	C26H28ClN3S
Molecular weight	450.04 g/mol
Num. heavy atoms	31
Num. arom. heavy atoms	17
Fraction Csp3	0.46
Num. rotatable bonds	6
Num. H-bond acceptors	2
Num. H-bond donors	0
Molar Refractivity	128.76
TPSA	56.01 Å²

Lipophilicity	
Log $P_{o/w}$ (iLOGP)	3.78
Log $P_{o/w}$ (XLOGP3)	6.87
Log $P_{o/w}$ (WLOGP)	6.59
Log $P_{o/w}$ (MLOGP)	6.03
Log $P_{o/w}$ (SILICOS-IT)	6.07
Consensus Log $P_{o/w}$	5.87

Water Solubility	
Log *S* (ESOL)	-6.97
Solubility	4.84e-05 mg/ml ; 1.08e-07 mol/l
Class	Poorly soluble
Log *S* (Ali)	-7.86
Solubility	6.26e-06 mg/ml ; 1.39e-08 mol/l
Class	Poorly soluble
Log *S* (SILICOS-IT)	-8.55
Solubility	1.27e-06 mg/ml ; 2.83e-09 mol/l
Class	Poorly soluble

Pharmacokinetics	
GI absorption	High
BBB permeant	No
P-gp substrate	Yes
CYP1A2 inhibitor	No
CYP2C19 inhibitor	No
CYP2C9 inhibitor	Yes
CYP2D6 inhibitor	Yes
CYP3A4 inhibitor	Yes
Log K_p (skin permeation)	-4.17 cm/s

Druglikeness	
Lipinski	Yes; 1 violation: MLOGP>4.15
Ghose	No; 1 violation: WLOGP>5.6
Veber	Yes
Egan	No; 1 violation: WLOGP>5.88
Muegge	No; 1 violation: XLOGP3>5
Bioavailability Score	0.55

Medicinal Chemistry	
PAINS	0 alert
Brenk	0 alert
Leadlikeness	No; 2 violations: MW>350, XLOGP3>3.5
Synthetic accessibility	5.87

Figure S9: Visual representation of the predicted ADME results of compound **D6** obtained from the online ADME prediction tool SwissADME.

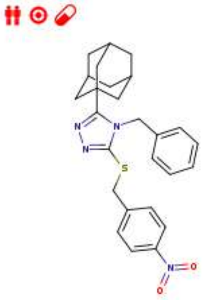

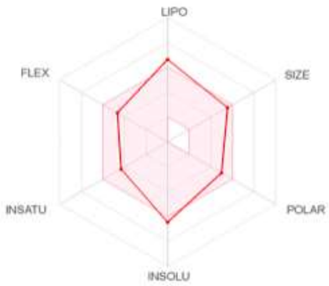

SMILES	O=N(=O)c1ccc(cc1)CSc1nnc(n1Cc1ccccc1)C12CC3CC(C2)CC(C1)C3

Physicochemical Properties	
Formula	C26H28N4O2S
Molecular weight	460.59 g/mol
Num. heavy atoms	33
Num. arom. heavy atoms	17
Fraction Csp3	0.46
Num. rotatable bonds	7
Num. H-bond acceptors	4
Num. H-bond donors	0
Molar Refractivity	132.57
TPSA	101.83 Å²

Lipophilicity	
Log $P_{o/w}$ (iLOGP)	3.78
Log $P_{o/w}$ (XLOGP3)	6.08
Log $P_{o/w}$ (WLOGP)	6.37
Log $P_{o/w}$ (MLOGP)	5.29
Log $P_{o/w}$ (SILICOS-IT)	3.65
Consensus Log $P_{o/w}$	5.03

Water Solubility	
Log *S* (ESOL)	-6.45
Solubility	1.65e-04 mg/ml ; 3.59e-07 mol/l
Class	Poorly soluble
Log *S* (Ali)	-8.00
Solubility	4.62e-06 mg/ml ; 1.00e-08 mol/l
Class	Poorly soluble
Log *S* (SILICOS-IT)	-7.78
Solubility	7.63e-06 mg/ml ; 1.66e-08 mol/l
Class	Poorly soluble

Pharmacokinetics	
GI absorption	Low
BBB permeant	No
P-gp substrate	Yes
CYP1A2 inhibitor	No
CYP2C19 inhibitor	Yes
CYP2C9 inhibitor	Yes
CYP2D6 inhibitor	Yes
CYP3A4 inhibitor	Yes
Log K_p (skin permeation)	-4.79 cm/s

Druglikeness	
Lipinski	Yes; 1 violation: MLOGP>4.15
Ghose	No; 2 violations: WLOGP>5.6, MR>130
Veber	Yes
Egan	No; 1 violation: WLOGP>5.88
Muegge	No; 1 violation: XLOGP3>5
Bioavailability Score	0.55

Medicinal Chemistry	
PAINS	0 alert
Brenk	1 alert: nitro_group
Leadlikeness	No; 2 violations: MW>350, XLOGP3>3.5
Synthetic accessibility	5.95

Figure S10: Visual representation of the predicted ADME results of compound D**7** obtained from the online ADME prediction tool SwissADME.

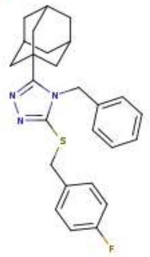

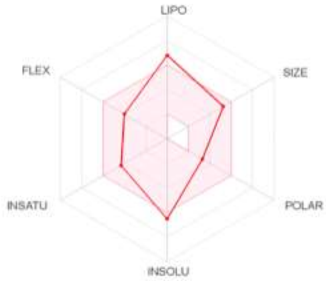

SMILES Fc1ccc(cc1)CSc1nnc(n1Cc1ccccc1)C12CC3CC(C2)CC(C1)C3

Physicochemical Properties	
Formula	C26H28FN3S
Molecular weight	433.58 g/mol
Num. heavy atoms	31
Num. arom. heavy atoms	17
Fraction Csp3	0.46
Num. rotatable bonds	6
Num. H-bond acceptors	3
Num. H-bond donors	0
Molar Refractivity	123.71
TPSA	56.01 Å²

Lipophilicity	
Log $P_{o/w}$ (iLOGP)	3.97
Log $P_{o/w}$ (XLOGP3)	6.35
Log $P_{o/w}$ (WLOGP)	6.49
Log $P_{o/w}$ (MLOGP)	5.93
Log $P_{o/w}$ (SILICOS-IT)	5.85
Consensus Log $P_{o/w}$	5.72

Water Solubility	
Log *S* (ESOL)	-6.54
Solubility	1.25e-04 mg/ml ; 2.89e-07 mol/l
Class	Poorly soluble
Log *S* (Ali)	-7.32
Solubility	2.09e-05 mg/ml ; 4.82e-08 mol/l
Class	Poorly soluble
Log *S* (SILICOS-IT)	-8.23
Solubility	2.56e-06 mg/ml ; 5.92e-09 mol/l
Class	Poorly soluble

Pharmacokinetics	
GI absorption	High
BBB permeant	No
P-gp substrate	Yes
CYP1A2 inhibitor	No
CYP2C19 inhibitor	No
CYP2C9 inhibitor	Yes
CYP2D6 inhibitor	Yes
CYP3A4 inhibitor	Yes
Log K_p (skin permeation)	-4.44 cm/s

Druglikeness	
Lipinski	Yes; 1 violation: MLOGP>4.15
Ghose	No; 1 violation: WLOGP>5.6
Veber	Yes
Egan	No; 1 violation: WLOGP>5.88
Muegge	No; 1 violation: XLOGP3>5
Bioavailability Score	0.55

Medicinal Chemistry	
PAINS	0 alert
Brenk	0 alert
Leadlikeness	No; 2 violations: MW>350, XLOGP3>3.5
Synthetic accessibility	5.88

Figure S11: Visual representation of the predicted ADME results of compound **D8** obtained from the online ADME prediction tool SwissADME.

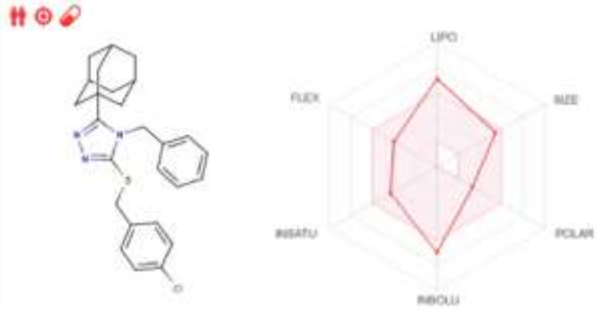

SMILES Clc1ccc(cc1)CSc1nnc(n1Cc1ccccc1)C12CC3CC(C2)CC(C1)C3

Physicochemical Properties	
Formula	C26H28ClN3S
Molecular weight	450.04 g/mol
Num. heavy atoms	31
Num. arom. heavy atoms	17
Fraction Csp3	0.46
Num. rotatable bonds	6
Num. H-bond acceptors	2
Num. H-bond donors	0
Molar Refractivity	128.76
TPSA	56.01 Å²

Lipophilicity	
Log $P_{o/w}$ (iLOGP)	4.17
Log $P_{o/w}$ (XLOGP3)	6.87
Log $P_{o/w}$ (WLOGP)	6.59
Log $P_{o/w}$ (MLOGP)	6.03
Log $P_{o/w}$ (SILICOS-IT)	6.07
Consensus Log $P_{o/w}$	5.95

Water Solubility	
Log *S* (ESOL)	-6.97
Solubility	4.84e-05 mg/ml ; 1.08e-07 mol/l
Class	Poorly soluble
Log *S* (Ali)	-7.86
Solubility	6.26e-06 mg/ml ; 1.39e-08 mol/l
Class	Poorly soluble
Log *S* (SILICOS-IT)	-8.55
Solubility	1.27e-06 mg/ml ; 2.83e-09 mol/l
Class	Poorly soluble

Pharmacokinetics	
GI absorption	High
BBB permeant	No
P-gp substrate	Yes
CYP1A2 inhibitor	No
CYP2C19 inhibitor	No
CYP2C9 inhibitor	Yes
CYP2D6 inhibitor	Yes
CYP3A4 inhibitor	Yes
Log K_p (skin permeation)	-4.17 cm/s

Druglikeness	
Lipinski	Yes; 1 violation: MLOGP>4.15
Ghose	No; 1 violation: WLOGP>5.6
Veber	Yes
Egan	No; 1 violation: WLOGP>5.88
Muegge	No; 1 violation: XLOGP3>5
Bioavailability Score	0.55

Medicinal Chemistry	
PAINS	0 alert
Brenk	0 alert
Leadlikeness	No; 2 violations: MW>350, XLOGP3>3.5
Synthetic accessibility	5.84

Figure S12: Visual representation of the predicted ADME results of compound **D9** obtained from the online ADME prediction tool SwissADME.

Table S20. Complete data list of PASS predictions for the activity spectrum of compound **1** (date of prediction: 2020-12-01).

**Pa**	**Pi**	**Activity name**
0,619	0,002	11-Beta-hydroxysteroid dehydrogenase inhibitor
0,610	0,002	11-Beta-hydroxysteroid dehydrogenase 1 inhibitor
0,596	0,009	Atherosclerosis treatment
0,573	0,026	Antiviral (Picornavirus)
0,531	0,017	Antiobesity
0,510	0,006	Antiparkinsonian, rigidity relieving
0,503	0,022	Antiviral (Influenza)
0,518	0,085	Kidney function stimulant
0,425	0,037	Antidiabetic
0,399	0,019	Antidiabetic (type 2)
0,388	0,026	PfA-M1 aminopeptidase inhibitor
0,369	0,009	Dual specificity phosphatase inhibitor
0,384	0,053	UGT2B12 substrate
0,342	0,020	Antiviral (Influenza A)
0,344	0,026	Calpain inhibitor
0,324	0,012	Calcium channel N-type blocker
0,289	0,009	Cytidine deaminase inhibitor
0,280	0,002	11-Beta-hydroxysteroid dehydrogenase 2 inhibitor
0,335	0,062	Antimycobacterial
0,324	0,071	Transcription factor STAT inhibitor
0,296	0,042	Thiol protease inhibitor
0,347	0,097	Alcohol O-acetyltransferase inhibitor
0,334	0,085	HMGCS2 expression enhancer
0,309	0,062	Transcription factor STAT3 inhibitor
0,320	0,074	Antiviral (Adenovirus)
0,309	0,065	Antituberculosic
0,266	0,029	Antidepressant, Imipramin-like
0,327	0,104	Polarisation stimulant
0,390	0,168	Fusarinine-C ornithinesterase inhibitor
0,238	0,022	GST M1-1 substrate
0,214	0,005	Antiacromegalic
0,246	0,037	Mcl-1 antagonist
0,304	0,103	Prion diseases treatment
0,242	0,043	Mucorpepsin inhibitor
0,312	0,115	CDK9/cyclin T1 inhibitor
0,259	0,064	GST P substrate
0,269	0,095	Prostate disorders treatment
0,236	0,062	GST P1-1 substrate
0,301	0,132	EIF4E expression inhibitor
0,174	0,010	Ca(v)2.2 blocker
0,186	0,022	Beta lactamase inhibitor
0,210	0,050	GABA B receptor agonist

0,231	0,083	Gestagen antagonist
0,348	0,200	Chymosin inhibitor
0,348	0,200	Acrocylindropepsin inhibitor
0,348	0,200	Saccharopepsin inhibitor
0,188	0,044	Calcium channel blocker
0,177	0,034	HCV NS3-helicase inhibitor
0,236	0,100	Aldosterone antagonist
0,211	0,081	Oryzin inhibitor
0,176	0,047	Antischistosomal
0,253	0,125	Neuropeptide Y2 antagonist
0,251	0,126	L-glutamate oxidase inhibitor
0,176	0,052	DNA directed RNA polymerase inhibitor
0,286	0,165	RNA-directed RNA polymerase inhibitor
0,125	0,004	CDC25B inhibitor
0,171	0,056	Alkaline phosphatase inhibitor
0,188	0,078	Tropinesterase inhibitor
0,183	0,074	Dual specificity phosphatase 1 inhibitor
0,118	0,010	Estradiol 17 beta dehydrogenase 2 inhibitor
0,301	0,200	Phospholipid-translocating ATPase inhibitor
0,112	0,014	Antiprotozoal (Histomonas)
0,194	0,096	Stroke treatment
0,216	0,119	ATP phosphoribosyltransferase inhibitor
0,312	0,218	5-O-(4-coumaroyl)-D-quinate 3'-monooxygenase inhibitor
0,269	0,178	(R)-6-hydroxynicotine oxidase inhibitor
0,202	0,116	Antibacterial
0,210	0,126	5 Hydroxytryptamine 1E antagonist
0,173	0,089	Antiparkinsonian, tremor relieving
0,203	0,119	Transcription factor inhibitor
0,207	0,124	Gamma-glutamyltransferase inhibitor
0,178	0,098	Cutinase inhibitor
0,295	0,216	Neurotransmitter uptake inhibitor
0,159	0,081	Paraoxonase substrate
0,115	0,039	Transglutaminase 2 inhibitor
0,322	0,247	Ubiquinol-cytochrome-c reductase inhibitor
0,284	0,209	Apyrase inhibitor
0,220	0,146	Cardiotonic
0,160	0,088	Acaricide
0,181	0,110	Carnitinamidase inhibitor
0,149	0,077	Thioredoxin reductase inhibitor
0,145	0,074	Hexokinase inhibitor
0,088	0,017	Protein-tyrosine phosphatase 2C inhibitor
0,207	0,136	ATPase stimulant
0,251	0,188	Thymidylate 5'-phosphatase inhibitor
0,261	0,199	Glucan endo-1,3-beta-D-glucosidase inhibitor
0,249	0,188	Muscular dystrophy treatment
0,200	0,140	Chloride channel activator

0,246	0,186	Benzoate-CoA ligase inhibitor
0,273	0,215	Phosphatidylcholine-retinol O-acyltransferase inhibitor
0,210	0,151	Plastoquinol-plastocyanin reductase inhibitor
0,197	0,140	CYP2C9 inhibitor
0,189	0,137	Lactose synthase inhibitor
0,169	0,117	Hyponitrite reductase inhibitor
0,209	0,157	Urethanase inhibitor
0,055	0,005	Equilibrative nucleoside transport protein 1 inhibitor
0,149	0,099	Inorganic diphosphatase inhibitor
0,194	0,144	CYP2B11 substrate
0,244	0,194	Insulysin inhibitor
0,179	0,130	Prolyl aminopeptidase inhibitor
0,125	0,079	Tardive dyskinesia treatment
0,080	0,038	Dysmenorrhea treatment
0,098	0,059	CDC25A inhibitor
0,087	0,050	Xanthine oxidase substrate
0,112	0,077	Somatostatin 2 agonist
0,124	0,090	Nicotinic alpha6 receptor agonist
0,109	0,075	Calcium channel (voltage-sensitive) blocker
0,181	0,149	Channel-conductance-controlling ATPase inhibitor
0,232	0,201	S-formylglutathione hydrolase inhibitor
0,126	0,095	GST T1-1 substrate
0,126	0,095	GST T substrate
0,037	0,006	Equilibrative nucleoside transport protein inhibitor
0,215	0,185	Lipoprotein lipase inhibitor
0,159	0,129	Polarisation inhibitor
0,266	0,236	Neurotransmitter antagonist
0,154	0,127	Antirickettsial
0,185	0,159	Antineoplastic (brain cancer)
0,297	0,273	Polyporopepsin inhibitor
0,201	0,177	Focal adhesion kinase 2 inhibitor
0,039	0,015	Methionyl aminopeptidase 2 inhibitor
0,044	0,022	Acetyl-CoA transferase 2 inhibitor
0,185	0,167	Pyruvate decarboxylase inhibitor
0,228	0,212	Cyclohexanone monooxygenase inhibitor
0,035	0,020	Sodium/glucose cotransporter 1 inhibitor
0,043	0,029	CXC chemokine 2 receptor antagonist
0,024	0,013	Antibiotic Oxacephem-like
0,215	0,205	Uterine relaxant
0,048	0,039	Purinergic P2Y agonist
0,048	0,040	Cyclin-dependent kinase 5 inhibitor
0,095	0,090	Alpha-pinene-oxide decyclase inhibitor
0,078	0,072	D-Ala-D-Ala ligase inhibitor
0,084	0,080	Thermomycolin inhibitor
0,159	0,157	Glucan 1,4-alpha-maltotetraohydrolase inhibitor
0,227	0,226	N-hydroxyarylamine O-acetyltransferase inhibitor

Table S21. Complete raw data list of PASS predictions for the activity spectrum of compound **2** (date of prediction: 2020-12-01).

Pa	Pi	Activity name
0,738	0,005	Antiobesity
0,687	0,005	Atherosclerosis treatment
0,673	0,002	11-Beta-hydroxysteroid dehydrogenase inhibitor
0,665	0,002	11-Beta-hydroxysteroid dehydrogenase 1 inhibitor
0,563	0,016	Antidiabetic
0,488	0,011	Antidiabetic (type 2)
0,515	0,088	Kidney function stimulant
0,405	0,020	Antiparkinsonian, rigidity relieving
0,378	0,009	Calcium channel N-type blocker
0,341	0,012	Dual specificity phosphatase inhibitor
0,321	0,005	Ca(v)2.2 blocker
0,304	0,002	11-Beta-hydroxysteroid dehydrogenase 2 inhibitor
0,304	0,048	PfA-M1 aminopeptidase inhibitor
0,296	0,044	Antiviral (Influenza A)
0,311	0,066	Neuropeptide Y2 antagonist
0,267	0,052	Thiol protease inhibitor
0,340	0,128	Diabetic neuropathy treatment
0,291	0,079	Prostate disorders treatment
0,294	0,083	Transcription factor STAT inhibitor
0,299	0,090	Antiviral (Influenza)
0,231	0,035	GABA B receptor agonist
0,256	0,067	Calpain inhibitor
0,193	0,007	Antiacromegalic
0,258	0,081	CYP2C9 inhibitor
0,213	0,042	Dual specificity phosphatase 1 inhibitor
0,323	0,153	Phosphatidylcholine-retinol O-acyltransferase inhibitor
0,339	0,172	Antiviral (Picornavirus)
0,343	0,189	5-O-(4-coumaroyl)-D-quinate 3'-monooxygenase inhibitor
0,180	0,029	Cytidine deaminase inhibitor
0,233	0,085	CYP3A4 inhibitor
0,205	0,057	Antidepressant, Imipramin-like
0,167	0,023	Narcolepsy treatment
0,246	0,107	Retinoprotector
0,138	0,007	Estradiol 17 beta dehydrogenase 2 inhibitor
0,190	0,068	Menstruation disorders treatment
0,256	0,133	Analgesic, non-opioid
0,151	0,029	Tardive dyskinesia treatment
0,174	0,054	DNA directed RNA polymerase inhibitor
0,122	0,004	CDC25B inhibitor
0,196	0,081	Gastric antisecretory
0,248	0,136	Serum-glucocorticoid regulated kinase 1 inhibitor
0,307	0,201	Neurotransmitter uptake inhibitor

0,213	0,109	Transcription factor inhibitor
0,276	0,173	Insulin promoter
0,140	0,048	HCV NS3-helicase inhibitor
0,148	0,057	Nicotinic alpha6 receptor agonist
0,271	0,186	Dementia treatment
0,150	0,068	Alkaline phosphatase inhibitor
0,115	0,037	CDC25A inhibitor
0,190	0,114	Tankyrase inhibitor
0,234	0,159	Antiviral (Adenovirus)
0,131	0,057	Beta lactamase inhibitor
0,202	0,129	Transcription factor STAT3 inhibitor
0,089	0,017	Protein-tyrosine phosphatase 2C inhibitor
0,156	0,088	Niemann-Pick C1-like 1 protein antagonist
0,092	0,024	Dysmenorrhea treatment
0,252	0,185	Insulysin inhibitor
0,143	0,076	Hexokinase inhibitor
0,301	0,245	Antineurotic
0,191	0,139	Arylmalonate decarboxylase inhibitor
0,117	0,069	Somatostatin 2 agonist
0,054	0,007	Acetyl-CoA transferase 2 inhibitor
0,101	0,055	Nicotinic alpha2beta4 receptor agonist
0,179	0,134	Glucan 1,4-alpha-maltotetraohydrolase inhibitor
0,149	0,110	GST M1-1 substrate
0,045	0,009	Methionyl aminopeptidase 2 inhibitor
0,157	0,122	GABA receptor agonist
0,203	0,169	Hypolipemic
0,203	0,172	All-trans-retinyl-palmitate hydrolase inhibitor
0,186	0,158	Albendazole monooxygenase inhibitor
0,039	0,013	Equilibrative nucleoside transport protein 1 inhibitor
0,051	0,024	Phospholipase A2 IIa inhibitor
0,186	0,161	N-formylmethionyl-peptidase inhibitor
0,250	0,226	Acetylgalactosaminyl-O-glycosyl-glycoprotein beta-1,3-N-acetylglucosaminyltransferase inhibitor
0,197	0,174	Cardiotonic
0,084	0,061	TRKA antagonist
0,037	0,016	Sodium/glucose cotransporter 1 inhibitor
0,214	0,195	Alkenylglycerophosphocholine hydrolase inhibitor
0,028	0,010	Antibiotic Oxacephem-like
0,054	0,039	Estradiol 17 beta dehydrogenase inhibitor
0,043	0,028	CXC chemokine 2 receptor antagonist
0,080	0,065	Tyrosine-protein kinase receptor antagonist
0,050	0,037	Purinergic P2Y agonist
0,046	0,037	Secretory phospholipase A2 inhibitor
0,025	0,016	Acetyl-CoA transferase 1 inhibitor
0,027	0,018	Equilibrative nucleoside transport protein inhibitor
0,152	0,144	Lipocortins synthesis antagonist

0,182	0,175	CYP2C3 substrate

Table S22. Complete list of PASS predictions for the activity spectrum of compound **3** (date of prediction: 2020-12-01).

**Pa**	**Pi**	**Activity name**
0,783	0,005	Antiobesity
0,676	0,002	11-Beta-hydroxysteroid dehydrogenase inhibitor
0,667	0,002	11-Beta-hydroxysteroid dehydrogenase 1 inhibitor
0,665	0,005	Atherosclerosis treatment
0,616	0,043	5-O-(4-coumaroyl)-D-quinate 3'-monooxygenase inhibitor
0,566	0,015	Antidiabetic
0,504	0,006	Antiparkinsonian, rigidity relieving
0,482	0,011	Antidiabetic (type 2)
0,488	0,056	Antiviral (Picornavirus)
0,515	0,088	Kidney function stimulant
0,471	0,060	Insulysin inhibitor
0,416	0,027	Neuropeptide Y2 antagonist
0,359	0,010	Dual specificity phosphatase inhibitor
0,338	0,005	GABA B receptor agonist
0,329	0,001	11-Beta-hydroxysteroid dehydrogenase 2 inhibitor
0,389	0,069	Insulin promoter
0,330	0,012	Calcium channel N-type blocker
0,381	0,063	Diabetic neuropathy treatment
0,344	0,036	PfA-M1 aminopeptidase inhibitor
0,322	0,036	Thiol protease inhibitor
0,349	0,064	Antiviral (Influenza)
0,271	0,005	Ca(v)2.2 blocker
0,284	0,023	Antidepressant, Imipramin-like
0,300	0,041	Antiviral (Influenza A)
0,287	0,049	Calpain inhibitor
0,300	0,080	Transcription factor STAT inhibitor
0,291	0,072	Transcription factor STAT3 inhibitor
0,275	0,069	CYP2C9 inhibitor
0,203	0,005	Antiacromegalic
0,224	0,034	Dual specificity phosphatase 1 inhibitor
0,209	0,021	Cytidine deaminase inhibitor
0,414	0,227	Phobic disorders treatment
0,387	0,204	Phosphatase inhibitor
0,272	0,093	Prostate disorders treatment
0,218	0,040	Menstruation disorders treatment
0,292	0,127	Antiseborrheic
0,241	0,078	CYP2C3 substrate
0,279	0,119	CYP2A8 substrate
0,198	0,038	DNA directed RNA polymerase inhibitor

0,238	0,079	CYP3A4 inhibitor
0,326	0,181	27-Hydroxycholesterol 7alpha-monooxygenase inhibitor
0,177	0,034	HCV NS3-helicase inhibitor
0,144	0,004	CDC25B inhibitor
0,188	0,049	Alkaline phosphatase inhibitor
0,282	0,151	Prion diseases treatment
0,324	0,196	Glycosylphosphatidylinositol phospholipase D inhibitor
0,126	0,009	Estradiol 17 beta dehydrogenase 2 inhibitor
0,218	0,105	Transcription factor inhibitor
0,295	0,185	Phosphatidylcholine-retinol O-acyltransferase inhibitor
0,281	0,172	RNA-directed RNA polymerase inhibitor
0,209	0,102	Glucagon-like peptide 1 agonist
0,307	0,201	Neurotransmitter uptake inhibitor
0,126	0,028	CDC25A inhibitor
0,111	0,013	Dysmenorrhea treatment
0,151	0,068	Hexokinase inhibitor
0,314	0,232	CYP2J2 substrate
0,185	0,104	Stroke treatment
0,225	0,145	Antimycobacterial
0,090	0,016	Protein-tyrosine phosphatase 2C inhibitor
0,261	0,188	EIF4E expression inhibitor
0,154	0,081	Tetrahydroxynaphthalene reductase inhibitor
0,219	0,148	CYP2C19 inhibitor
0,177	0,106	Pediculicide
0,202	0,136	Chloride channel activator
0,198	0,135	X-methyl-His dipeptidase inhibitor
0,159	0,097	Myeloblastin inhibitor
0,131	0,069	Glycine receptor agonist
0,226	0,165	Analgesic, non-opioid
0,124	0,064	Beta lactamase inhibitor
0,129	0,069	Tardive dyskinesia treatment
0,262	0,208	Venombin AB inhibitor
0,131	0,079	Nicotinic alpha6 receptor agonist
0,209	0,159	Cardiotonic
0,165	0,117	Prostaglandin E1 antagonist
0,117	0,069	Somatostatin 2 agonist
0,115	0,068	Renal failure treatment
0,179	0,134	Glucan 1,4-alpha-maltotetraohydrolase inhibitor
0,283	0,238	Complement factor D inhibitor
0,206	0,163	L-glutamate oxidase inhibitor
0,269	0,227	Gastrin inhibitor
0,209	0,169	CYP2C29 substrate
0,127	0,087	Mcl-1 antagonist
0,097	0,060	Heme oxygenase inhibitor
0,099	0,063	Cathepsin G inhibitor
0,284	0,249	Trans-acenaphthene-1,2-diol dehydrogenase inhibitor

0,140	0,108	Acaricide
0,156	0,124	GABA receptor agonist
0,203	0,172	All-trans-retinyl-palmitate hydrolase inhibitor
0,275	0,246	CYP2J substrate
0,168	0,138	Diuretic
0,304	0,275	Nicotinic alpha4beta4 receptor agonist
0,041	0,013	Sodium/glucose cotransporter 1 inhibitor
0,186	0,158	Albendazole monooxygenase inhibitor
0,226	0,199	JAK2 expression inhibitor
0,186	0,161	N-formylmethionyl-peptidase inhibitor
0,039	0,014	Methionyl aminopeptidase 2 inhibitor
0,250	0,226	Acetylgalactosaminyl-O-glycosyl-glycoprotein beta-1,3-N-acetylglucosaminyltransferase inhibitor
0,038	0,014	Equilibrative nucleoside transport protein 1 inhibitor
0,214	0,195	Alkenylglycerophosphocholine hydrolase inhibitor
0,209	0,190	CYP17 inhibitor
0,290	0,274	Antiviral (Rhinovirus)
0,278	0,263	Mucomembranous protector
0,051	0,036	Purinergic P2Y agonist
0,105	0,091	Glutamate release inhibitor
0,026	0,011	Antibiotic Oxacephem-like
0,135	0,121	Antiparkinsonian, tremor relieving
0,053	0,040	Estradiol 17 beta dehydrogenase inhibitor
0,077	0,067	UDP-N-acetylmuramate dehydrogenase inhibitor
0,042	0,032	CXC chemokine 2 receptor antagonist
0,199	0,189	FMO3 substrate
0,229	0,221	HMGCS2 expression enhancer
0,048	0,040	Cyclin-dependent kinase 5 inhibitor
0,191	0,183	Hypolipemic
0,071	0,064	Scytalone dehydratase inhibitor
0,026	0,019	Equilibrative nucleoside transport protein inhibitor
0,152	0,146	Gastric antisecretory
0,110	0,104	DELTA24-sterol reductase inhibitor
0,067	0,065	Preterm labor treatment
0,170	0,170	Saluretic
